# Journey into Bone Models: A Review

**DOI:** 10.3390/genes9050247

**Published:** 2018-05-10

**Authors:** Julia Scheinpflug, Moritz Pfeiffenberger, Alexandra Damerau, Franziska Schwarz, Martin Textor, Annemarie Lang, Frank Schulze

**Affiliations:** 1 German Federal Institute for Risk Assessment (BfR), German Centre for the Protection of Laboratory Animals (Bf3R),10589 Berlin, Germany; julia.scheinpflug@bfr.bund.de (J.S.); franziskawschwarz@googlemail.com (F.S.); martin.textor@bfr.bund.de (M.T.); 2 Charité—Universitätsmedizin Berlin, corporate member of Freie Universität Berlin, Humboldt-Universität zu Berlin and Berlin Institute of Health, Department of Rheumatology and Clinical Immunology, 10117 Berlin, Germany; moritz.pfeiffenberger@charite.de (M.P.); alexandra.damerau@charite.de(A.D.); annemarie.lang@charite.de (A.L.); 3 German Rheumatism Research Centre (DRFZ) Berlin, a Leibniz Institute, 10117 Berlin, Germany

**Keywords:** bone, in vitro models, biomaterials, bioreactors, organ-on-a-chip

## Abstract

Bone is a complex tissue with a variety of functions, such as providing mechanical stability for locomotion, protection of the inner organs, mineral homeostasis and haematopoiesis. To fulfil these diverse roles in the human body, bone consists of a multitude of different cells and an extracellular matrix that is mechanically stable, yet flexible at the same time. Unlike most tissues, bone is under constant renewal facilitated by a coordinated interaction of bone-forming and bone-resorbing cells. It is thus challenging to recreate bone in its complexity in vitro and most current models rather focus on certain aspects of bone biology that are of relevance for the research question addressed. In addition, animal models are still regarded as the gold-standard in the context of bone biology and pathology, especially for the development of novel treatment strategies. However, species-specific differences impede the translation of findings from animal models to humans. The current review summarizes and discusses the latest developments in bone tissue engineering and organoid culture including suitable cell sources, extracellular matrices and microfluidic bioreactor systems. With available technology in mind, a best possible bone model will be hypothesized. Furthermore, the future need and application of such a complex model will be discussed.

## 1. Introduction

The musculoskeletal system determines the body’s shape and is mandatory for unrestricted locomotion in vertebrates. Here, bone is the key component in conveying stability, force distribution and protection of the inner organs. Bone pathologies can cause immobility, support dependence and inflexibility that can be accompanied by pain and do therefore represent a tremendous loss of life quality and a socioeconomic burden for the health care system [1,2]. Innovative basic and translational research approaches pursue the goal of prevention and protection. The current gold-standard in preclinical drug screening and proof-of-concept studies for innovate treatments is the use of animal models. Nevertheless, interspecies differences in physiology and metabolism are thought to be the main reason for the limited transferability of findings from animal studies to humans, as exemplified by the high failure rates of potential new therapies in clinical trials, although based on promising results from animal studies [3,4]. Hence, there is a great need to facilitate a shift in biomedical research approaches towards an elevated relevance for human physiology. During the past decades, traditional in vitro cell culture systems have been revised and improved in order to reflect human physiology in a more relevant manner. This development was mainly driven by the triumph of regenerative medicine that was facilitated through several improvements in tissue engineering. These include the isolation and large-scale expansion of primary cells or the use of biocompatible scaffold materials for creating three-dimensional (3D) artificial tissues as grafting material. Such advances also enabled the development of sophisticated biomaterials, bioreactors and microfluidic platforms that can be used in the context of innovative human-relevant in vitro systems as alternatives or predictive support to animal testing [5,6,7,8,9,10].

Bone is a rather complex tissue with a multitude of functions and consists of several different cell types while the extracellular matrix (ECM) is a composite material of an organic and an inorganic phase [11]. Although well vascularized, the oxygen levels in bone range from 6% down to 1% and is well established that a low oxygen environment, also termed physioxia, is crucial for the hematopoietic stem cells (HSCs) phenotype [12]. Related to its role in vertebrate locomotion, bone is under constant mechanical load that influences local cell behaviour [13]. Thus, complete recreation of bone physiology is challenging and requires co-culture of multiple cell types and a bioreactor system capable of perfusion and mechanical actuation.

This review aims to provide an overview on current developments and approaches to model bone physiology and pathology in vitro. Since monolayer cultures are of limited value when complex interactions of organs or tissues need to be investigated, 3D but not two-dimensional (2D) models will be in the focus of this review. Details will be provided on different scaffold-free models, carrier systems (scaffolds or hydrogels), processing and cultivation systems (3D print, bioreactors, organ-on-a-chip). For this, we not only draw information from in vitro bone models but also from the field of clinical bone grafts and implants that are meant to substitute critical size defects or facilitate targeted drug delivery. This approach is taken since it originates from the authors experience, that the wealth of information generated in clinical studies can be of high value in in vitro models and vice versa.

Following the summary of current approaches to generate bone models or bone-like grafts, we will discuss their particular strengths and limitations and speculate how current models might be improved towards a higher physiological relevance. Although summarizing studies from different fields, where requirements and limitations vary, we want to discuss and hypothesize a best possible in vitro bone model for basic research, drug development and toxicology.

## 2. Bone Physiology

Bone is a connective tissue that acts as a supportive component within the musculoskeletal system. This tissue is subject to a permanent, dynamic process of degradation of old, and formation of new, bone—a process termed remodeling—that is thought to be an adaptive response to mechanical load [14]. Furthermore, remodeling prevents accumulation of micro damages that can lead to fractures similar to the process of fatigue, known as a problem in material sciences [15]. Bone consists of an ECM that is a composite material and a multitude of specialized cell types with osteocytes (OCTs) being the most prevalent one. This ECM consists of approximately 60% inorganic components, 30% organic matrix, lipids and water [11]. Hydroxyapatite (HA), the mineralized part of bone, is the inorganic component composed of calcium and phosphorous and is responsible for the high mechanical stability of the ECM [16]. The HA crystals provide mechanical rigidity and load-bearing properties to bone whereas the organic matrix provides elasticity and flexibility. The organic matrix mainly consists of collagen I, which accounts for 85–90% of total bone protein [17].

**Figure 1:**
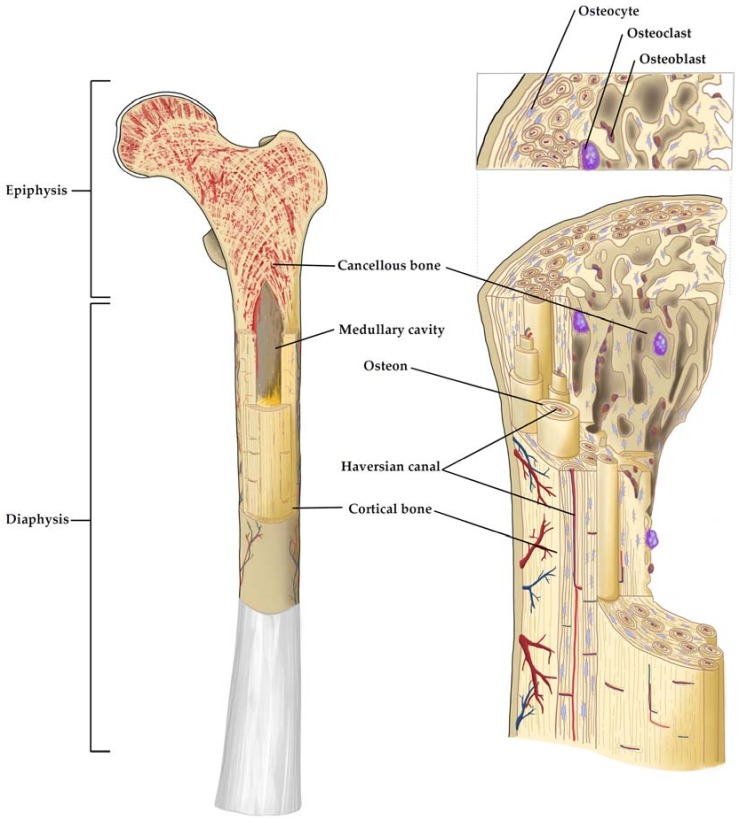
Bone Anatomy. A schematic drawing of the most profound anatomical features of bone, providing a cross section through cortical and cancellous bone while indicating the sites where the respective cells can be found.

This matrix composition is common throughout the two different histological types of bone tissue termed cortical and cancellous bone. Cortical bone has a dense ordered structure, while cancellous bone is less compact and has an irregular structure. These differences in their density and porosity result in different mechanical properties. In the diaphysis of long bones and the surface of flat bones, dense cortical bone provides resistance to torsion and bending, while cancellous bone provides mechanical flexibility and occurs at the epiphysis and medullary cavity of long bones and at the inside of flat bones. The main functional unit of cortical bone is the osteon. Multiple layers of osteoblasts (OBs) and OCTs are located around the Haversian canal that is situated in the centre of the osteon and contains blood and nerve vessels (Figure 1). The Haversian canals are interconnected by Volkmann's canals. On its outer surface, cortical bone is covered by the periosteum while the inner surface is covered by the endosteum that serves as a boundary between the cortical bone and the cancellous bone. In cancellous bone, the main functional unit is the trabecula that aligns according to the mechanical load bone encounters [18]. Bone marrow can be found in the cavities of cancellous bone and can be divided into the red active hematopoietic marrow and yellow inactive fat marrow. Red marrow is predominant during embryogenesis and is continuously converted to yellow bone marrow during skeletal maturation. The niche for HSCs that give rise to all blood and immune cells can be found in red bone marrow and is defined by complex interactions with stromal and vascular cell populations [19]. Furthermore, low oxygen levels within the bone marrow are a key signaling factor for the resident HSCs and are thought to be vital for stem cell expansion while maintaining the correct phenotype [12].

Osteoblasts, the bone building cells, are of mesenchymal origin and generate the osteoid, which is the unmineralized organic fraction of the bone matrix, by secreting collagen I, osteopontin, bone sialoprotein II and osteocalcin [20]. The secreted osteoid is then subsequently mineralized. Osteoblasts have a cuboidal morphology with a round, basal nucleus and basophilic cytoplasm. In the final phase of bone formation, OBs eventually become entrapped by the bone matrix they created. They then turn into a quiescent state and become terminally differentiated OCTs with a significant reduced ability to form new bone, while those OBs not entrapped by their matrix will undergo apoptosis [21].

Osteocytes are mature, non-proliferating cells that are 10 times more abundant in bone than OBs and account for >90% of total bone cells. They have a star-shaped morphology and communicate through canaliculi, gap junctions and dendritic offshoots [22]. Furthermore, they are involved in the regulation of OB and OCT activity and there is increasing evidence that OCTs can act as mechanosensors through their dendritic processes within bone and thus govern the remodeling process in response to altered mechanical loading. In contrast to the previously mentioned cell types, multinucleated osteoclasts (OCs) are derived from hematopoietic mononuclear precursor cells (monocyte–macrophage lineage) and are responsible for bone resorption, which enables the dynamic bone remodeling processes [23,24].

The formation of flat and long bones during embryogenesis is facilitated through the distinctive processes of intramembranous and endochondral ossification, respectively. Both processes are excellently reviewed in detail elsewhere and will only be briefly explained in the current review [25,26]. During intramembranous ossification, OBs condense and proliferate in the ossification centre, followed by direct secretion of the osteoid’ components and subsequent mineralization. The flat bone precursor is then vascularized and remodelled. The process of endochondral ossification starts with the condensation and proliferation of chondrocytes that form a cartilage anlage. Chondrocytes that get embedded in ECM by this process start to get hypertrophic, secrete matrix metalloproteinases and eventually undergo apoptosis, thereby creating a sponge-like structure. The cartilage matrix is then vascularized and mineralized by recruited endothelial precursors and OBs in the primary ossification centre. The medullary cavity is then formed by resorption and remodeling of the newly formed bone tissue. Secondary ossification centres form at the bones’ diaphysis for longitudinal growth at the growth plates. This continuous process of new cartilage formation, mineralization and remodeling between the primary and secondary ossification centre is ongoing until adulthood is reached, which is signified by the complete ossification of the growth plate.

## 3. Cell Types and Sources for Bone Tissue Engineering 

Since bone is comprised of a multitude of different cell types, bone models should incorporate a diverse repertoire of cells. For the purpose of studying fracture healing and endochondral bone formation, mesenchymal stromal cells (MSCs) are thought to be of key importance to evaluate the intermediate states of soft callus formation during fracture healing and endochondral ossification during embryogenesis, respectively. Therefore, MSCs provide a suitable precursor for the generation of bone organoids. Further incorporation of OBs and OCs in a multicellular approach might be of advantage, due to their significance in formation and remodeling of hard fracture callus and woven bone [27]. In addition to these unicellular and/or multicellular approaches, which are based on purified single-cell populations, multicellular concentrates such as bone marrow aspirate concentrate (BMAC) and microfragmented adipose tissue could also be used for in vitro bone modeling. Bone marrow aspirate concentrate contains all resident bone marrow cells except red blood cells that are separated during the concentration process and therefore all relevant cell types necessary for in vitro bone formation [28]. In addition to the already mentioned MSCs, OBs and HSCs, these also encompass endothelial precursors that are essential for the vascularization of the tissue. Nonetheless, BMAC remains an undefined cell concentrate, which would mean that any organoid produced would be unique in the exact cell composition, thus hampering reproducibility. Further, economic issues such as limited accessibility and limited yield of BMAC result in a restricted number of organoids that can be generated, compared to the number of organoids that can be produced by in vitro expansion of single cell populations. Similar to BMAC, micro fragmented adipose tissue contains numerous cells capable of secreting a variety of bioactive molecules which can elicit angiogenic, antifibrotic, antiapoptotic, antimicrobial and immunomodulatory responses in the target tissue [29]. However, with the exception of MSCs and endothelial progenitors, these cell populations do not represent those found in bone.

### 3.1. Mesenchymal Stromal Cells—The Work Horse in Bone Research

Over the past two decades, numerous publications have been published on MSCs isolated from various tissue sources [10,30,31,32,33,34,35,36]. In contrast to human embryonic and foetal stem cells, the utilization of which might be accompanied by ethical considerations, human MSCs can be isolated from adult tissue and are thus considered to be a suitable cell source for in vitro model systems and tissue engineering. MSCs were first isolated from whole bone marrow of rodents by plastic adherence selection in 1970 by Friedenstein et al. [37]. In addition to their undifferentiated state, bone marrow-derived MSCs (BM-MSCs) have a limited ability for self-renewal and exhibit a multi-lineage differentiation potential into OBs, chondrocytes and adipocytes. The advantages in availability, multipotency and the potential for allogenic application due to their immune-evasive nature, gave rise to the broad application of MSCs in bone research and beyond. MSCs can be isolated and expanded with relative ease using standard cell culture techniques. In vitro aging is nonetheless a limiting factor in terms of expansion for therapeutic approaches, since MSCs undergo rapid senescence after a few passages [38]. Due to their differentiation potential, MSCs were early suspected to possess regenerative capabilities. Yet, studies show limited evidence for in vivo differentiation of MSCs after transplantation. It is thus still a matter of debate what mode of action facilitates the regenerative potential of MSCs. More attention has come to the paracrine machinery of MSCs and their implication for the modulation of tissue environments and cells [39]. When compared to other primary cell types, MSCs are more versatile in regard of their multipotency and their paracrine machinery.

However, MSCs can exhibit functional differences depending on their tissue source, resulting in a site-specific phenotype for MSCs [40,41]. For example, orofacial BM-MSCs proliferate faster, show a higher alkaline phosphatase (ALP) activity and an increased in vitro calcium accumulation as compared to iliac crest BM-MSCs after osteogenic stimulation. In contrast, iliac crest BM-MSCs form more compact bone in vivo and show a greater response to osteogenic and adipogenic induction in vivo and in vitro [40].

Since the availability of human BM-MSCs is limited as a result of a very painful retrieval procedure or the reliance on bone marrow that is discarded in the course of orthopaedic surgeries, other isolation sources from a variety of tissues, such as adipose tissue [42], umbilical cord blood [32,35,36,43], peripheral blood [44], skin [31,45], teeth [46,47], cartilage [34], pancreas [48] and liver [49] have been studied and characterized during the last decades. Adipose tissue-derived MSCs (AT-MSCs) and umbilical cord-derived MSCs (UC-MSCs) can be isolated from adipose tissue and umbilical cord, respectively. Both tissues seem to be an attractive source to obtain sufficient cell yields since the material used for isolation is clinically available in huge quantities and is usually discarded. All above described MSCs can be characterized by the common stem cell features, as described for BM-MSCs but differ in properties such as colony formation ability, proliferation, the multi-lineage differentiation capacity but also the proteome and transcriptome, depending on the cell source used [31,32,33]. A major advantage of UC-MSCs and AT-MSCs is the less invasive procedure to obtain tissue material for cell isolation [32,35]. Furthermore, UC-MSCs are less aged than MSCs from other sources, thus these cells have a higher proliferation rate and can be maintained in culture for more passages [32]. However, the isolation efficiency for UC-MSCs is distinctly lower compared to BM-MSCs and AT-MSCs [36]. Moreover, the isolated UC-MSCs exhibit a diminished colony formation ability and poorly differentiate towards the adipogenic lineage when compared to MSCs from other sources [32,36]. Isolated AT-MSCs show better cell yields, faster cell proliferation and expansion and a higher colony formation ability when compared to BM-MSCs [30]. With regard to the multilineage differentiation potential, a wealth of data on the differences between BM-MSCs and AT-MSCs has been published. Kern et al. [35] and Rebellato et al. [36] postulate that there are no differences between AT-MSCs and BM-MSCs in terms of adipogenic and osteogenic differentiation, which is partially backed by Noel and co-workers who showed that the adipogenic differentiation potential is the same for both AT-MSCs and BM-MSCs [33]. In contrast, Peng et al. [34] have performed a comparative analysis of MSCs from bone marrow and adipose tissue and demonstrated that MSCs differentiate source specifically, that is BM-MSCs differentiate better towards the osteogenic while AT-MSCs show better differentiation towards the adipogenic lineage. These findings were confirmed by Al-Nbaheen et al. [31], who demonstrated that BM-MSCs differentiate better towards the osteogenic lineage than AT-MSCs. In summary, the results of these studies indicate that the differentiation potential of MSCs is a function of their tissue of origin. The tissue specific differences between BM-MSCs and AT-MSCs are underlined by their expression of genes such as Wnt and mitogen-activated protein kinase (MAPK) and genes related to fatty acid metabolism or cell communication [31,33]. These differences related to the cell’s origin should be kept in mind when using MSCs for tissue engineering of bone. Bone marrow-derived MSCs might be of favour due to their source and the related high osteogenic differentiation capacity, yet the source material for AT-MSCs can be acquired with relative ease and in comparably higher quantities.

### 3.2 Bone-Forming and -Resorbing Cells, Cell Lines and Stem Cells

Interestingly, MSCs and not OBs or OCTs are primarily used in bone research. With OBs being the primary bone forming cell and OCTs those that comprise over 90% of all bone cells, the main actors in bone physiology seem underrepresented in related research as the focus is evidently drifted towards MSCs [50]. A major disadvantage of OCTs is the difficult isolation procedure with multiple digestion and decalcification steps [51] that might lead to cell alterations, as reported for similar isolation procedures for OBs. In addition to MSCs, which can be differentiated into OBs, terminally differentiated cells can also be isolated from bone tissue and expanded in cell culture for in vitro applications [52]. Further, circulating OBs can be isolated from peripheral blood [53,54,55]. Bone marrow-derived OBs can also be obtained via enzymatic tissue digestion, which might have a negative impact on ALP activity and/or mineral deposition levels. Therefore, OBs are rather isolated by a simple outgrowth procedure from bone fragments onto the plastic surface of a suitable culture vessel. Primary cells are known to exhibit phenotypic heterogeneity caused by donor variability. Therefore, OBs of species where specific strains are available (murine, bovine, ovine) are often used for in vitro studies to prevent donor-related heterogeneity by minimizing variability. Although interindividual differences are more controllable between animals from a certain strain, the osteogenic phenotype is still a function of age, gender and environment (reviewed in [56]). In a human setting, primary material for cell isolation is usually derived from long bones during orthopaedic surgery. The need for therapeutic intervention in the musculoskeletal system is at its highest in the elderly population. Thus, most cell material is isolated from aged donors that already suffer from concomitant diseases rather than from young and healthy donors. It is well established that not only age, gender and site of isolation but also medications and concomitant disease of donors can have profound effects on isolated cells [57]. This might lead to problems considering tissue engineering and developmental approaches if biased by elderly cell sources and their peculiarities, which is also a challenge for BM-MSCs isolation. On the other hand, for therapeutic approaches and diseases modeling these circumstances should be considered as an advantage or opportunity rather than a drawback. In any case, phenotypic characterisation of the isolated cells, for example their proliferation rate, metabolic activity and matrix mineralization can help to determine donor-dependent heterogeneity. This information can be used to group isolated cells according to the phenotype relevant for experimentation. For example, experiments that investigate the effect of a given compound on cell growth should use cells from donors with a similar proliferation rate to counteract donor-dependent heterogeneity. In addition, information about concomitant diseases, medications, acquisition procedure and site of isolation can be of value for the characterisation and classification of donor derived material as these parameters can all affect the cellular phenotype.

Hematopoietic stem cells and their progeny OCs might be considered as additions to existing bone models, if remodeling, healing processes and cellular interactions in the HSC niche are being investigated [58,59,60]. A further example for the importance to incorporate immune cells is the negative impact of T-cells on bone regeneration and impaired healing outcome [61].

Human embryonic stem cells (ESCs) can give rise to cells from all germ layers and therefore also all bone cells but have the biggest drawback due to ethical considerations, availability and great demand on cell culture techniques. Regulatory limitations are usually a knockout criterion for most researches, yet the potential of ESCs remains enormous, especially considering developmental research [62].

Bridging primary cells and cell lines, induced pluripotent stem cells (iPSCs) are in between as they are pluripotent cells derived from primary cells and do not occur in nature. Induced pluripotent stem cells are still restricted to basic research primarily due to the possibility of incomplete differentiation that might cause malignancies after transplantation, which further upstream, leads to translational issues. Future improvements of reprogramming techniques might abolish these flaws and also lead to higher differentiation frequencies and efficiencies. In addition, possible alterations of expression profiles, pathways and phenotypes always have to be kept in mind. Besides of these concerns, iPSCs have great potential in models for developmental research or specific diseases, for which currently no cell source exists or the use of ESCs is restricted due to ethical issues [10,63].

Cell lines on the other hand (e.g., MC3T3-E1, SaOs2, MG-63 or hTERT MSCs), allow for expansion in high quantities, with strong conformity and great reproducibility, yet it has been shown that they pose an inadequate replacement for primary cells by various studies. Drawbacks include malignancies, non-physiological behaviour and reduced differential potential, which raises questions regarding the translational value of cell lines [56,64,65,66]. In summary, the most promising cell source for bone models and translational research are primary human cells due to their non-malignant nature, differentiation state and functional phenotype.

## 4. Approaches for Creating a Bone-Like Organoid

### 4.1. Scaffold-Free Self-Organisation in Spheroids

To gather a better glance into osteogenic processes and for mimicking the in vivo situation more properly, research draws the focus on organoids such as spheroid cultures, since they represent a rather simple approach to culture cells in a 3D construct that might also allow for self-organization of cells. Spheroids are simple organoids with a rounded morphology and provide a 3D environment that enables cell–cell contacts [67,68]. They are relatively easy to produce, handle and offer potential for medium- to high-throughput applications. Therefore, spheroids are an increasingly attractive approach in organoid generation, most often to mimic tissues like liver [69,70], brain [71], tumour models [72,73] but also with regard to in vitro bone models [74,75,76,77]. Generally, the differentiation potential of MSCs is evidently enhanced in 3D spheroid cultures [68,78,79].

Thus, Gurumurthy et al. were able to show superior osteogenic differentiation of human AT-MSCs in a 3D spheroid culture when compared to monolayer cultivation [80]. Different spheroid-forming techniques have been developed to establish scaffold-free 3D models for osteogenesis, ranging from liquid suspension culture, hanging droplet suspension towards non-adhesive culture plates. Here, multitier plates have shown to be the most efficient, well controlled and easiest to handle method [74]. In a complex multicellular spheroid system, de Baros et al. demonstrated matrix deposition and expression of osteogenic genes, similar to the in vivo situation [81]. There have been also models implementing an osteoblastic–osteoclastic–endothelial cell co-culture system, thus accomplishing osteogenesis and bone-resorbing OC activity [82].

For mimicking the in vivo situation more adequately, new approaches also draw their focus towards the combination of osteogenic and endothelial cell types, particularly for the generation of vascularized bone. When seeding human umbilical vein endothelial cells (HUVECs) and human OBs in a collagen matrix to produce a 3D spheroidal model both, osteogenic differentiation and tube-like structures, were found [83]. Till date, the effects of endothelial cell types on osteogenic processes remain elusive [84]. With the focus on synergistic relationships between endothelial and osteoblastic cells, early co-culture models demonstrated an upregulation of osteogenic markers like ALP [85,86]. Steiner et al. [87] and Leszczynska et al. [88] were able to demonstrate a positive synergistic effect on the proliferation rate of human OBs and MSCs accompanied by an elevated ALP and collagen expression when co-cultured with HUVECs. Contrarily, the results of Guillotin et al. [89] and Xue et al. [90] indicate a significant downregulation of osteogenic marker genes such as runt-related factor-2 (RUNX2) and osteocalcin in osteoprogenitor cells or MSCs. In summary, working with spheroids enables osteogenic differentiation in vitro in a 3D setting thereby modeling processes such as bone development or bone healing to a certain amount.

### 4.2. Scaffold-Based Approaches for Bone Tissue Engineering

The most common approach to create artificial bone involves the combination of a scaffold and bone cells followed by static or dynamic cultivation in a culture vessel or bioreactor system, respectively. Besides positively influencing cell differentiation and proliferation, the scaffold is expected to support cell colonization and migration, thus acting as the main stimulus on the development of the desired tissue [91]. The requirements for the design of an ideal scaffold for bone tissue engineering have been comprehensively reviewed elsewhere [7,92,93,94,95]. In the current review, scaffold-related criteria that are important for generating physiologic relevant human bone models in vitro are of special interest. The ideal scaffold should imitate the native bone ECM as close as possible. Additionally, the following characteristics should be considered: (i) sufficient biocompatibility to promote cell attachment and survival [95]; (ii) suitable surface properties that trigger cell differentiation and proliferation [96]; (iii) adequate mechanical properties to mimic the mechanical properties of the tissue of interest [97]; (iv) a porous structure that permits cell reorganization and vascularization [96]; and (v) good biodegradability [98]. The biocompatibility, that is the ability of the scaffold to support normal cellular activity including molecular signaling without causing any unwanted physiological response, is one of the most important concerns. Subsequently, the ideal scaffold for the generation of bone organoids needs to be osteoinductive and osteoconductive. Osteoinduction describes the capability of the scaffold to promote the differentiation of undifferentiated or precursor cells towards bone-forming cells. In contrast, osteoconduction defines the ability of a scaffold to facilitate bone growth including cell adherence, proliferation and ECM formation [99,100]. Closely related to osteoinductivity and conductivity are the surface properties of each scaffold—which are determined by surface chemistry and structure—that affect cell attachment and migration [94,101]. For this, the surface of a given material can be fitted with different charges or proteins and peptides that are recognized by cell surface receptors (reviewed in [92]). In addition to surface properties, the mechanical properties of a given scaffold should be taken into account. Since the biomechanical features (compressive strength, stiffness and elasticity) of natural bone differ greatly between the cancellous (spongiosa) to cortical (compact) bone, it is quite problematic to design the “universal” bone scaffold. As comprehensively reviewed in [92,102], cancellous bone has super elastic mechanical properties with a Young’s modulus in the range of 0.1 to 2 GPa and a compressive strength of between 2 and 20 MPa. In contrast, the Young’s modulus and compressive strength of the cortical bone are 15–20 GPa and 100–200 MPa, respectively. The overall porosity of bone also affects its mechanical properties. The porosity of cancellous bone is 50–90%, while an environment with 3–12% porosity can be found in cortical bone [103]. However, it is well investigated that an increase in porosity and pore size of artificial scaffolds reduces their mechanical strength [104]. In the literature, a minimum pore size of 100 μm in diameter is described to be required in scaffolds to ensure a sufficient oxygen supply (hypoxic conditions) and thus induce endochondral ossification in vitro. Pore sizes around 300 μm are recommended as the optimum for the production of artificial bone due to the initiation of direct osteogenesis and subsequent vascularization [105]. Furthermore, complete degradability of the scaffold would be desirable to facilitate remodeling. In this regard, it should be noted that the process of bioresorption should be variable and controllable, for both in vivo and in vitro studies. In summary, the proper design of porous scaffolds with an ideal composition, including optimized surface and mechanical properties and associated degradation is one of the key challenges for the successful implementation of bone models. The natural ECM of bone consists of around 30% organic material (proteins and polysaccharides), 60% inorganic minerals, lipids and water, while the exact composition strongly dependents on parameters such as age, gender and bone type [11]. A variety of natural and synthetic components have been tested for their applicability as bone scaffolds. These include: (i) natural biomaterials such as decellularized tissue explants and purified ECM components [106,107]; (ii) biopolymers that can be subdivided into natural polymers such as gelatin, alginate, fibrin, collagen, chitosan, hyaluronic acid [108,109,110], and synthetic polymers such as poly-(lactide) (PL), poly-(glycolide) (PG), poly-(lactide-co-glycolide) (PLG), poly-(lactide-co-lactide) (PLL), poly-(caprolactone) (PCL) [111,112,113]; (iii) bioceramics such as bioglass, calcium (apatite), bicalcium and tricalcium phosphates and corals [114,115]; (iv) metals and their alloys such as cobalt–chromium alloys, stainless steel, aluminium and titanium alloys and [116,117]; (v) combinations of different material groups like natural polymers/ceramics, synthetic polymers/ceramics or metal/ceramics [118,119,120]. According to the proposed scaffold criteria, frequently used material types were selected and evaluated in this review. Since the inorganic bone mineral is a crystalline salt of calcium and phosphorous, bioceramics—especially calcium phosphate (CaP)-based materials and their composites—have been widely studied for their utility. The most common CaPs used are HA, β-tricalcium phosphate (β-TCP) and bicalcium phosphates (BCPs) made from HA-β-TCP, HA and β-TCP composites [121,122]. Therefore, in vitro model systems using these materials as scaffolds are highlighted and discussed below. Furthermore, an overview of the published studies of the last decade using other scaffold materials, cell sources and in vivo models is given in Supplementary Table S1.

#### 4.2.1. Hydroxyapatite and its Composites as Scaffold Materials in Human Bone Models

As mentioned above, 60% of bone matrix are an inorganic crystalline phase which is basically HA (Ca_10_(PO_4_)_6_(OH)_2_) [123]. The characteristics of HA have already been extensively reviewed in [124,125,126] and will be briefly summarized. Hydroxyapatite is commercially available as granules or blocks with different porosity and pore sizes (micropores: 2–8 µm, macropores 250–350 μm) resulting in varying mechanical properties. Furthermore, with a calcium/phosphate ratio of 1.67, HA is the most stable but also poorly soluble CaP. Although this ratio results in good osteoconductivity, the poor solubility leads to a lower osteoinductivity and degradation rate. A promising method to enhance osteoinductivity and biodegradability is the modification of HA with ionic substitutes, amino acid sequences such as arginine–glycine–aspartic acid (RGD) peptides or whole protein structures. In addition, HA composites that combine the benefits of HA and a given composite partner are used frequently. These upgrades of HA increase its solubility thus enhancing its bioactivity and, ultimately, cell differentiation and scaffold mineralization (reviewed in [125]). In this context, Meskinfam et al. studied the morphological, chemical, mechanical properties and in vitro interactions of rat MSCs with untreated polyurethane (PU) foam scaffolds and treated biomineralized (surface activation with HA) PU scaffolds [127]. The results show that treatment of PU foam results in a better biocompatibility, as demonstrated by a good cell to matrix attachment and proliferation rate. Further, mineralization leads to improved mechanical properties compared to pure PU foam. Nevertheless, the mechanical properties of the tested scaffolds, with a Young’s modulus of 13.75 kPa for untreated PU and values between 15.67 and 25.40 kPa for treated PU, are too low to reproduce the elasticity of natural cancellous bone [127]. In another study, Mitra et al. recently published their data comparing pure PLG scaffolds with a composite scaffold made of HA and PLG that meets the above-mentioned criteria for a suitable bone scaffold—osteoconductivity, biodegradability, porosity and mechanical properties [99]. For this, human MSCs were seeded in PLG and PLG–HA scaffolds under static and dynamic conditions in a perfusion bioreactor to compare both cell differentiation and the effect of different pore sizes of PLG–HA scaffolds (small: 125–300 µm, middle: 300–500 µm, large: 500–850 µm) on the cell distribution within the scaffold. MSC differentiation was enhanced in PLG–HA scaffolds as compared to pure PLG scaffolds when cells were cultured under perfusion. The size of the scaffold pores correlated directly with the efficiency of cell seeding under static culture conditions, that is the cells were better distributed in scaffolds with a larger pore size. In contrast, cells were similarly well dispersed within scaffolds irrespective of pore sizes when cultured under perfusion [99]. In summary, the 3D PLG–HA scaffold in the perfusion bioreactor from Mitra et al. provides a model system that appears to be suitable for in vitro bone development. Instead of applying mechanical stimulation through compression, the scaffolds were perfused only in the present model. Nevertheless, they demonstrated that HA is the component of the scaffold composite that appears to be responsible for MSC differentiation towards the osteogenic linage [99]. A similar approach was pursued by Zhou et al. who investigated PL, PL–collagen I, PL–HA and PL–HA–collagen I scaffolds populated with cells form a murine osteoblastic cell line (MC3T3-E1) [128]. In contrast to Mitra et al. [99], Zhou et al. [128] pointed out that biocomposites containing collagen—which is part of the natural ECM in bone—are more efficient than the combination of HA and PL alone. The cells cultured in PL–HA–collagen I scaffolds showed enhanced adhesion, spreading, proliferation, differentiation, mineralization and expression of osteogenic genes when compared to other scaffolds tested.

In contrast, Mao et al. replaced collagen with ethyl cellulose (EC) and investigated the structural, functional and mechanical properties of a PL–HA–EC scaffold with varying HA levels. The results showed that PL–HA–EC scaffolds exhibited optimal structural, functional and mechanical properties at 20 wt % HA loading level. With a porosity of about 84% and a pore size between 150 and 250 μm, the PL–HA_(20wt %)_–EC scaffold achieves a Young’s modulus of 35.21 ± 3.17 MPa and a maximum compressive yield strength of 1.57 ± 0.09 MPa. While the Young’s modulus is lower than that of natural cancellous bone, the compressive strength reported is indeed in the correct range (1–100 MPa). Scaffolds without and with wt % HA contents other than 20 wt % showed a decrease in both the compressive strength and the Young's modulus of the scaffold. However, since Mao et al. did not colonize the scaffolds with cells, no biocompatibility assessment can be made and thus the evaluation of this model system as a suitable in vitro bone model is rather difficult [91]. Summarizing both studies it can be concluded that a combination of PL/HA and a protein or polysaccharide component seems to represent an effective scaffold variant in the context of bone tissue engineering. In addition to the stimulation of cell attachment, proliferation and differentiation, the introduction of HA can also help to mimic mechanical properties that are very similar to those of natural bone. In both studies, the scaffold degradation rate was slowed down in vitro when HA was added to PL–EC (56 days) [91] and PL–collagen (80 days) [128]. In contrast to these studies, Duan et al. [129] focused on different scaffold–medium combinations to define the best conditions for ideal ossification in a bioreactor [129]. Therefore, they maintained AT-MSCs in commercially available bovine collagen I, bovine collagen I–β-TCP and bovine collagen-I–β-TCP–HA scaffolds and compared different media variants (osteogenic or stromal and a combination of both). Similar to other studies, it was demonstrated that the addition of HA to existing scaffolds leads to the best in vitro osteogenesis, closely followed by pure bovine collagen I scaffolds. These results indicate that osteogenesis is mediated by different signaling pathways through collagen I- and HA-containing scaffolds. However, no mechanical load was applied and the collagen I was of bovine origin, which is not ideal for mimicking a human in vitro model system. In summary, the results of the described in vitro model systems indicate the benefits of HA in combination with collagen I as scaffold matrices. By using these two materials, the cell porosity, the pore size but also the mechanical properties can be adapted to physiological conditions, enabling a biocompatible model similar to the in vivo environment for bone cells and their precursors.

#### 4.2.2. Tricalcium Phosphate and Its Composites as Scaffolds in Human Bone Models

In recent years, the development of CaP-based bone replacement materials has increased significantly because of their great chemical and crystallographic similarity to the natural apatite. As mentioned before, more than 60% of the natural bone ECM consists of an inorganic crystalline phase comprising calcium salt and phosphoric acid, analogously represented in the bone replacement material TCP. Tricalcium phosphate represents a ceramic with the chemical formula Ca_3_(PO_4_)_2_ [130]. There are three polymorphs constituting synthetic TCP, the rhombohedral β-TCP which is used most frequently and two more thermostable forms. All forms have a tetrahedral phosphate centre linked through oxygen to the calcium ions. Pure phase TCP is already being used as a scaffold in bone regeneration in most dental implant procedures and is available as block, paste or granules with different porosity and pore sizes in a range of 5–500 µm resulting in a great variety of possible applications [130]. The interconnected pores enable cells to infiltrate the scaffold and provide an optimal microenvironment for OB adhesion and proliferation. β-TCP is biodegradable and osteoconductive which means that this porous ceramic can facilitate physiologic bone growth. The biomaterial itself has no osteoinductive properties, which mandates the addition of osteogenic factors while the sole use of calcium phosphate ceramic materials is limited. Bernhardt et al. already characterized Cerasorb M^®^ and Bio-Oss^®^, which supports cell adhesion, proliferation and osteogenic differentiation. A perfusion system promotes more uniform cell distribution and can improve the osteogenic differentiation of human MSCs seeded onto β-TCP [131]. Another promising method to increase cell adhesion and osteogenic differentiation is the modification of β-TCP with chemical substitutes, amino acid sequences or protein structures. In this context, Deng et al. studied vascularization and bone formation by using β-TCP doped with different ratios of calcium silicate (CS) and co-culturing HUVECs and human BM-MSCs [132]. These experiments demonstrated that no chemical reactions occurred between both components resulting in good cell viability for 5% CS-β-TCP. Additionally, cell proliferation was inhibited and cell viability was reduced as the proportion of CS exceeded 10%. Other studies showed a higher activity of osteogenic markers in the presence of 5% CS compared to pure β-TCP. In conclusion, the 5% CS–β-TCP combination was biocompatible and enhanced osteogenic differentiation of human MSCs. Furthermore, HUVEC angiogenesis was stimulated as determined in vitro by tube formation as a key step of angiogenesis and in vivo by micro- Computer Tomography measurements after subcutaneous implantation in mice [132]. Due to the importance of vascularization in bone formation, Kang et al. developed a vascularized bone graft by using a biomimetic cell sheet engineered periosteum and a porous β-TCP scaffold [133]. For this, human MSCs were cultured in a dish until they reached confluency. Subsequently, a cell suspension of HUVECs was seeded and co-cultured with the undifferentiated MSC cell sheet to develop a prevascularized cell sheet. In addition, an osteogenic MSC sheet was developed by cultivation in osteogenic medium, then placed on the prevascularized cell sheet and wrapped around the β-TCP scaffold. The results of this in vitro study demonstrated that HUVECs formed a robust network, which implied that the cell sheet provided a suitable microenvironment for cell migration and growth. Hence, the prevascularized biomimetic periosteum promotes vascularization and osteogenesis in vitro and in vivo. This was assessed via haematoxylin and eosin examination confirming that β-TCP with a prevascularized cell sheet formed more blood vessels in comparison to the β-TCP scaffold without a prevascularized cell sheet in vivo [133]. The induction of osteogenesis can be enhanced by controlled drug release. Therefore, β-TCP was loaded with Simvastatin, a lipid-lowering medication and the release was controlled by the addition of an outer apatite layer. In summary, this might be a possibility to ensure a sustained release of drugs that enhance the repair and healing of bone fractures [134]. In contrast, ZnO and β-TCP were introduced into a 58S bioactive glass scaffold to study bioactivity and biodegradability [135]. Bioactive glass is available in different compositions, while 58S signifies a mixture of 60 wt % SiO_2_, 36 wt % CaO and 4 wt % P_2_O_5_. Here, human osteogenic sarcoma cells (MG63 cells) were transplanted on a pre-wetted scaffold under static conditions. The 58S glass–β-TCP–ZnO composite scaffold promotes better cell attachment, proliferation and improved degradability in comparison to pure 58s glass, whereas the initial compressive strength of the 58S glass scaffolds and 58s glass–β-TCP–ZnO composite scaffolds were 30.28 ± 2.79 MPa and 23.66 ± 3.9 MPa. This demonstrates that β-TCP and ZnO are viable enhancers to improve the bioactivity and degradability in scaffolds for bone tissue engineering [135]. Moreover, the combination of different scaffold materials can enhance their biological properties. Ceramic particles composed of 60% HA and 40% TCP, mimicking the mineral part of bone, were pre-incubated with serum-containing medium and then seeded with human primary OBs which differentiated into OCTs in the absence of differentiation agents. Nevertheless, the expression of OCT-specific markers increased in 3D culture compared to 2D culture [136]. In contrast to the studies already discussed, Zima et al. [137] compared the effects of several components like calcite or HA doped with Mg^2+^, CO_3_^2−^ or Ag^+^, alginate and chitosan on the properties of α-TCP to optimize setting times, biochemical stability, bioresorption and biocompatibility. Results showed that HA doped with Mg^2+^ and CO_3_^2−^ or a combination of silver, calcite and reactive α-TCP enhanced cell viability in comparison to pure α-TCP. The advantage of combining components with high reactive α-TCP is promising tool the field of bone engineering [137].

### 4.3. Hydrogel-Based Bone Models

Since the pioneering work of Wichterle and Lim in 1960 [138], hydrogels have been of great interest in the field of biomaterials, tissue engineering and 3D printing [139,140] due to their potential application as 3D matrices [141]. Hydrogels are hydrophilic, cross-linked networks of polymers [142]. They have structural similarities to macromolecular-based components in the body and are regarded as biocompatible [143]. While they can be made of any water soluble polymer, there is a variety of chemical compositions and physical properties [141]. Due to their particular properties, hydrogels are commonly used for many purposes such as (i) matrices for tissue engineering, (ii) regenerative medicine, and (iii) cellular immobilization [8,144,145]. Hydrogels can be produced from either natural or synthetic-derived polymers. Fibrin, gelatin, hyaluronic acid, fibroin, alginate and collagen belong to the naturally derived polymer hydrogels whereas polyethylene glycol (PEG), peptides and methacrylate rank among synthetic-based hydrogels. Hydrogels are versatile since they can be loaded with drugs, growth factors and/or cultured with cells promoting cellular activation, differentiation and distribution. The advantages and disadvantages of the use of this material for bone tissue engineering are listed in Table 1.

#### 4.3.1. Naturally Derived Polymer Hydrogels

The natural protein fibrin is often used in combination with other biomaterials as a coating agent for other scaffold materials, beads or injectable hydrogels. Park et al. studied the feasibility of osteogenic differentiation of human MSCs in a 3D construct made of fibrin mixed with bone morphogenetic protein (BMP)-2 loaded nanoparticles [146]. It is well established that BMP-2 induces osteogenic differentiation of MSCs. In accordance with this, the complex of fibrin and BMP-2 loaded nanoparticles leads to a higher osteoinduction in human MSCs compared to fibrin only. Interestingly, the use of BMP-2 conjugated nanoparticles yielded better results when compared to loading the fibrin hydrogel with native BMP-2. Other studies investigated the biological bone formation activity of BMP-2 by incorporating it into a gelatin hydrogel [147], or by using a gelatin based hydrogel that contains PLGA microspheres [148]. Thus, demonstrating that proteins like BMP-2 could be incorporated into hydrogels for improved bone formation in vitro. The naturally based polymer gelatin is a mixture of denatured collagens and can be used to produce hydrogels that can be further modified with various factors. Yet, depending on the hydrogel’s material properties and the way of factor delivery, the sustained release over time varies [146,149,150]. Hyaluronic acid is one of the major components of the ECM in connective and neural or epithelial tissues where it acts as a pivotal mediator of cell motility [151]. Cross-linked, hyaluronic acid–based hydrogels have been used to release BMP, resulting in superior osteogenesis [152,153]. In vivo studies demonstrated enhanced bone formation by using hyaluronic acid–based hydrogel in combination with human MSCs or BMP [153,154].

#### 4.3.2. Synthetic Polymer Hydrogels

Synthetic-based hydrogels, like the hydrophilic polymer PEG, are used to study their suitability for bone tissue engineering. Since PEG polymers do not promote cell adhesion, it has to be modulated by the addition of short peptides or an alteration of the molecular structure of the PEG itself [155,156]. The cell adhesion peptide RGD can be incorporated into PEG hydrogels thereby promoting a dose-dependent increase in cell adhesion and osteogenesis of MSCs [156]. Peled et al. developed a synthetic hydrogel made out of PEG conjugated to natural fibrinogen constituents that induces osteogenesis in and around the hydrogel implant in vivo, which is mediated through a sustained release of fibrinogen fragments [157]. In vivo studies demonstrate good biocompatibility, biodegradability and capacity of guided bone regeneration by using a three-component hydrogel composite. The developed hydrogel composite consists of poly(ethylene glycol)–poly(ε-caprolactone)–poly(ethylene glycol) (PECE) with incorporated amounts of nano-HA and collagen [158].

Hydrogels can even be doped with nanodiamonds (NDs), so-called nanocomposite hydrogels. The NDs can then act as a 3D scaffold for drug delivery to promote the osteogenic differentiation of MSCs. Pacelli et al. were able to demonstrate that the integration of NDs with dexamethasone in a hydrogel comprising photocrosslinkable gelatin methacrylamide is feasible [140]. This formulation allowed for a higher retention of dexamethasone over time, resulting in an increased expression of osteogenic-specific markers. This suggests that conventional hydrogels can be modified with conjugated NDs to develop a novel platform for bone tissue engineering.

#### 4.3.3. Scaffold–Hydrogels Hybrids

Other promising classes of biomaterials are hybrid scaffolds that show high potential in the field of bone tissue engineering such as thermo-sensitive chitosan hydrogels incorporated into 3D-printed PCL, which is well suited for cultivation with BM-MSCs. It is biocompatible and promotes osteogenic differentiation. Dong et al. demonstrated a stronger osteogenesis and matrix formation for rabbit MSCs grown in hybrid and chitosan hydrogels after 2-weeks in vitro cultivation [139]. Dhivya et al. prepared a hydrogel containing zinc-doped chitosan-nano-HA-β-glycerophosphate and demonstrated its potential with respect to bone formation without any toxic effect on MSCs in vitro and in vivo [141]. The addition of nano-HA resulted in an enhanced osteogenesis. It has recently been shown that it is possible to print a chitosan hydrogel and its composites with bone-like HA and cells, all in one formulation [159]. Here, the addition of HA resulted in increased cell proliferation and osteogenic differentiation. To optimize these hybrid scaffold systems, they can be conjugated with human recombinant BMP-2 through a biotin-streptavidin link. Igwe et al. created a hybrid scaffold consisting of nano-HA and poly(85lactide-co-15glycolide) and demonstrated its osteoinductive potential [160]. By combining this hybrid scaffold with human recombinant BMP-2 conjugated (arginine–alanine–aspartic acid–alanine)_4_ (RADA-16) peptide hydrogel, they fabricated a system with osteoinductive and weight-bearing features that could be used for treatment of critical-sized bone defects [160].

### 4.4. Bead-Based Tissue Engineering

A promising but less explored approach in tissue engineering is the use of polymeric beads to build up a 3D scaffold with a defined microarchitecture that allows for good cell migration. Furthermore, beads could function as a depot or delivery matrix for a controlled substance release within the scaffold material by facilitating a time dependent release as a function of surface pore size [161]. Agarwal et al. built up a 3D implant by closely stacking layers of hexagonal arranged packs of calcium alginate beads together. This microstructure leads to hypoxic conditions inside the construct and a pore size that enables cell migration, resulting in osteogenic differentiation of human MSCs [162]. Using implants that are made of hexagonal alginate bead packs allows for the controlled release of various substances like the antibiotic metronidazole against *Escherichia coli* or vascular endothelial growth factor (VEGF) which induces angiogenesis in a mice model. Alginate hydrogels containing cell-instructive materials that promote attachment are of interest as potential cell carriers in bone tissue engineering. Bhat et al. demonstrated that the presence of engineered ECM components on microbeads in alginate hydrogels promotes cell adhesion and osteogenic differentiation of MSCs without relying on cell-adhesive peptides [163]. The use of alginate beads doped with BMP-2 and platelet-rich factors leads to a sustained release that promotes cell proliferation and osteogenic differentiation in a dose-dependent manner. Platelet rich plasma can be easily isolated and further processed but suffers from a limited storage life that leads to early decomposition of signaling factors [164]. Beads can also be made out of bioactive ceramics such as HA and TCP. The advantages of combining both materials include the great mechanical strength and tissue adhesive properties of HA on the one hand and the high bioadsorbable properties of TCP on the other hand [165].

### 4.5. 3D Printing

During the advent of additive manufacturing, the potential of 3D printing techniques in the context of bone was explored early. First attempts aimed to generate scaffolds that mimic the chemical and biomechanical characteristics of bone [166]. These approaches, however, require sintering of the deposited material to achieve the desired stability of the constructs and are therefore not suited to incorporate cells in the printing process. Yet, generating cell free scaffolds as fitted implants through 3D print remains a promising approach in reconstructive surgery of bone [167]. For tissue engineering, bioprinting techniques such as inkjet writing (IW), extrusion printing (EP), laser-assisted forward transfer (LIFT) and stereolithography (SLA) are suitable since they allow the integration of living cells [168]. These methods are excellently reviewed in [166,169] and will not be discussed in depth here in favor of bioprinting in the context of engineering cellularized bone tissue. In theory, bioprinting can be employed for the reproducible generation of organoids, as it allows for the generation of specific structural features and the precise deposition of cells. Furthermore, it is possible to include vascularization in the organoid from the beginning, thus improving the exchange of oxygen, nutrients and metabolites. The most common method for bioprinting bone is EP as it allows for the use of hydrogels with varying viscosities and high cell densities [170,171,172,173]. One drawback in EP is the deposition process that is facilitated through mechanical extrusion of the bioink through a nozzle, thereby creating high shear forces that can negatively influence cell viability, especially for stem cells. Extrusion printing represents a robust and relatively simple bioprinting technique with the clear advantage of using a wide range of hydrogel-based bioink formulations. Due to their mechanical properties, hydrogels are not suitable for generating larger voids or hollow spaces since layer-by-layer dispositioning would result in collapse of structural features. Therefore, sacrificial materials like the poloxamere F-127 might be introduced to allow for printing hollow fibre structures such as vessel lumen for enhanced perfusion of the organoid or subsequent vascularization [174,175]. Although this allows for the bioprinting of more complex structures, the introduction of a sacrificial material might introduce challenges on its own. These include an increase of complexity in the printing process itself due to ongoing material exchange that requires multiple nozzles. However, the simultaneous use of different cell-laden and sacrificial inks was successfully demonstrated by Shim et al., emphasizing that the required engineering solutions are available for multi-nozzle 3D printing [176]. The sacrificial material needs to be biocompatible and should be printable under the same conditions as the employed bioinks, thus limiting the range of materials available [177]. Aside from EP, LIFT was also employed for bioprinting of bone [178,179]. Laser-assisted forward transfer has a higher resolution and is not associated with high shear forces for the cells, usually resulting in higher cell viability during the printing process. In addition, the bioinks used for LIFT have no restraints regarding viscosity as they do not need to be extruded from a nozzle. Compared to EP, the LIFT technique is far more complex and expensive thus limiting its routine application for most labs. For bioprinting bone, bioinks containing nano-HA have been employed in the context of LIFT [178,179]. In one study, Keriquel et al. [179] directly printed into critical size defects of animals using LIFT, thus closing the defect in bone tissue and demonstrating the versatility and applicability of this approach. Most of the above mentioned bioinks were used as carriers for primary cells and cell lines alike and have been shown to be cytocompatible while allowing for osteogenesis and, in some cases, also vasculogenesis.

## 5. Beyond the Dish Culturing Bone Models under Controllable Conditions 

### 5.1. Bioreactors for Culturing Bone

As described earlier, bone is a highly vascularized multicellular tissue with an ECM that is a composite material. Further, bone is a hypoxic tissue with oxygen gradients that govern the identity of stem cell populations and it is under constant mechanical load due to locomotion of the body. While the biological characteristics of bone can be mimicked in a tissue engineered construct or organoid, the physical parameters such as hypoxia and mechanical load need suitable bioreactor systems to be employed.

Bioreactors for culturing bone are needed for basic research or for culturing transplantable tissue constructs. When bone grafts are generated, the focus lies on providing a graft that can be biological incomplete but has the correct mechanical properties for implantation in critical size osseous defects. Here, the cellular composition of bone is meant to be reached by host ingrowth not by seeding in all different cell types upfront. If added, vast amounts of cells are needed to generate tissue engineered bone constructs in a suitable size range. Thus, classical bioreactors for the rapid and cost-effective expansion of cells were also used for the culture of bone cells and are reviewed extensively elsewhere [9]. In brief, the expansion of bone cells in high volumes can be facilitated in rotating wall reactors or spinner flasks. Mechanical forces are present as shear stress in these systems through perturbation of the culture medium. Although the suitability of these systems for the expansion of bone cells was shown, their main focus lies on the generation of a sufficient number of cells for tissue engineering rather than culturing tissue engineered organoids under mechanical load or hypoxia. Other attempts to expand or pre-differentiate bone cells such as OBs and MSCs in 3D scaffolds use reactor systems that are capable of perfusing the construct or additionally apply cyclic compression. Here, the boundaries between tissue engineering and translational research overlap regarding the applicability of the respective systems. For research questions, physiologic bone models are needed and the different systems suitable for this purpose will be the focus in this review section. A summary of the discussed bioreactor systems can be found in Supplementary Table S2.

One major hurdle in the in vitro culture of large organoids or scaffold-based grafts is the lack of vasculature and hence a limited diffusion of nutrients, metabolites and oxygen in and out of the constructs. Therefore, perfusion is needed for every larger organoid or graft to prevent formation of necrotic regions. Attempts to produce tissue engineered grafts for the treatment of critical-size bone defects use systems capable of perfusion culture. The most straight forward implementation of this insight is the use of a tube-like bioreactor with unidirectional flow [180]. Yet, this approach introduces gradients of nutrients and oxygen throughout the construct in the direction of flow, thereby creating heterogeneous distribution of cells resulting in non-uniform graft properties. To circumvent this problem, Wendt et al. introduced a perfusion culture system for cylindrical HA ceramic scaffolds that allowed for a uniform distribution of oxygen and nutrients within the graft leading to a heterogeneous distribution of cells [181]. In their system, the culture media is oscillating between two glass columns arranged as a U-tube, thereby perfusing two grafts bi-directionally. The system was subsequently used to investigate optimal seeding regime and ratio for multicellular bone grafts and the introduction of vasculature [182,183]. Grayson et al. used a cylindrical bioreactor that allowed for perfusion of anatomically shaped bone grafts through several inputs [184]. The most sophisticated design in terms of perfusion is introduced by Schmelzer et al. who use a hollow-fibre reactor originally designed as a liver bioreactor. Here, oxygen and different media are introduced into a foamy scaffold through a large number of intersecting hollow fibres to ensure optimal perfusion [185]. These perfusion approaches help to provide the tissue engineered bone constructs with oxygen and nutrients in a uniform way, while control over oxygen saturation in the media allows for the establishment of physiologic hypoxia. It needs to be mentioned that maintaining a defined oxygen level is a dynamic process since cells would consume oxygen as a function of their number and metabolic activity that can change over time. Further, oxygen might diffuse in and out the bioreactor setup according to the materials used. Thus, a reliable method to dynamically monitor and control oxygen levels in the culture media is of need.

Another parameter that needs to be included in bioreactor systems to increase their physiological relevance for bone is the application of mechanical forces. The skeletons’ main functions include the provision of mechanical support for the body itself, the protection of the inner organs and locomotion. During body movement, forces applied to the skeleton result in alterations concerning direct strain, shear stress and hydrostatic pressure experienced by bone cells. Mechanical forces are therefore constantly present in bone due to locomotion and it is well proven that this has a profound impact on cellular function [13,18,186]. In this regard, a number of proposed bioreactor systems for bone include the application of mechanical forces to either generate better developed grafts or models with an elevated physiologic relevance. Shear stress can be applied by perfusing the organoid, while direct strain and hydrostatic pressure are the result of compression due to mechanical load. The application of mechanical load was therefore implemented in different systems such as the one proposed by Matziolis et al. [187]. Here, the organoid is cultured in a tube-like reactor that can be housed in an incubator and allows for cyclic compression through pneumatic actuation. The reactor was rather designed for investigating the effect of cyclic mechanical stimulation on tissue engineered bone grafts than being used for establishing a physiologic bone model [188]. Yet, it served as a successful demonstration of the beneficial effects of mechanical load in the context of 3D in vitro bone formation. In this regard, the bioreactor model used by Kleinhans et al. was further modified to allow cyclic compression while perfusing the tissue construct [189]. Since bioreactor experiments are rather expensive regarding time and cost, parallelization can help to reduce these expenses per experiment while the overall throughput is enhanced. In this regard, Hoffmann and colleagues have developed a system that allows for perfusion and application of compressive forces in a bioreactor system that can run four units in parallel [190]. The force needed for longitudinal compression of a given material to a certain extend (usually expressed in percent of the original size) depends on the object’s stiffness that is described by the Young’s modulus. This parameter is a material property, however in biological systems such as tissue engineered organoids the Young’s modulus changes over time due to cellular activity such as ECM deposition and mineralization [191]. Thus, determining the organoids Young’s modulus over time is favourable if changes in stiffness due to biological activity need to be investigated. Furthermore, to keep the rate of compressive strain on a bone organoid constant over time, the applied mechanical load needs to be adapted to the changes in the Young’s modulus due to cellular activity. This needs ongoing determination of the Young’s modulus and dynamic adjustment of the applied compressive force rather than keeping it constant over time. For this, Jagozinski et al. have used a modified version of a bioreactor system that was designed for the culture of liver and allows for the determination of the applied force and compression by determining the subsequent change in the organoids height [192]. Regarding the determination of mechanical load and displacement, a more compact system was designed by Petersen et al. and successfully used to investigate the impact of mechanical load on BMP pathway signaling [191,193]. Although these systems allow for the investigation of material properties under cell culture conditions in unprecedented detail, their overall setup does not allow for mid- or high- throughput applications. To increase throughput while maintaining capabilities for perfusion and mechanical load, Richards et al. used exchangeable perfusion chambers that can be supplied with media through a 12-channel perfusion pump. The perfusion chambers themselves are designed in a way that allows for the transmission of compression forces when placed in a separate device [194]. The unit for the transmission and quantification of compressive forces also allows for the exact quantification of displacement and is therefore suitable for the ongoing determination of the organoid’s Young’s modulus [195]. Another more exotic type of bioreactor uses magnetic fields for the contact-free transduction of mechanical forces, an approach that eliminates the risk of contamination through movable parts needed for application of mechanical loads [196,197]. This approach however relies on the use of magnetic micro- and nanoparticles that might pose an unwanted component in the organoid itself due to possible side effects on the cells. Moreover, the spatial distribution of magnetic nanoparticles can change over time towards a more heterogeneous pattern thus changing the distribution of forces throughout the organoid [198]. None of the mentioned groups have tried to implement hypoxic conditions into their systems although at least the bioreactor of Peterson et al. would have the capability to do so by using a silicon rubber membrane for gas exchange in a low oxygen environment [191]. Future bioreactors should also provide the ability to determine and control oxygen levels in the reactor itself and thereby add another important physiologic parameter for the culture of bone organoids.

### 5.2. Microfluidics and the Next Generation Bone Bioreactor

A current development in the field of bioreactor technology encompasses the use of microfluidic systems for the recreation of organ function or aspects thereof, a concept named organ-on-a-chip (OoC). These microphysiological systems allow for the establishment of tissue-specific parameters such as mechanical strains, oxygen levels and the co-culture of different cell populations in 2D or 3D and can be regarded as miniaturized bioreactor systems [199,200]. Consequently, the wealth of information generated with classical bioreactor systems can be a solid foundation for developing smaller microfluidic OoCs.

**Figure 2:**
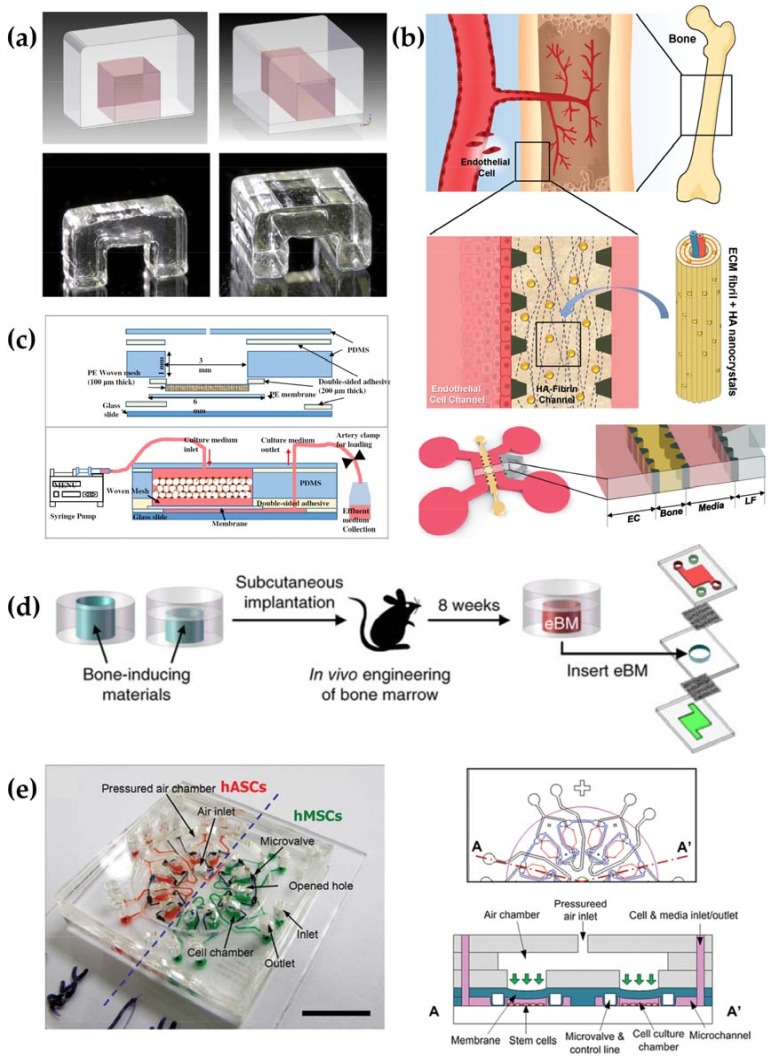
Different microfluidic systems for modeling a respective physiologic aspect of bone. (**a**) A polymethylmethacrylate (PMMA) cage was employed as a mould for a hydrogel-based model to investigate the effect of different oxygen levels on vascularization. Reprinted from [206] with permission from Elsevier. (**b**) The process of vascularization in bone matrix is modelled on a polydimethylsiloxane (PDMS) chip that uses posts to separate the different compartments while allowing for ingrowth of blood vessels. Reproduced from [205] with permission of the Royal society of Chemistry. (**c**) Beads are packed with osteocytes to mimic the canaliculi network found in bone and to allow for controlled perfusion. Reprinted from [207] with permission from Elsevier. (**d**) The bone organoid is formed ectopic in an animal prior cultivation in a PDMS-based perfusion chamber. Reprinted from [203] with permission from Nature/Springer/Palgrave. (**e**) Pneumatic actuation allows for the simultaneous application of mechanical forces (stretching) on mesenchymal stromal cells (MSCs) from different origins. Reprinted from [208] under the Creative Commons Attribution License.

A number of diverse OoC systems that rely on different materials and techniques for manufacture were proposed within recent years and are thoroughly summarized elsewhere [6,200,201]. However, when compared to published OoC systems such as lung, liver or kidney, bone appears to be underrepresented as indicated by the comparably low number of proposed systems [6,202]. Published microfluidic systems in the context of bone have clearly defined purposes such as recreating the bone marrow niche, recreating the 3D network of OCTs, modelling vasculogenesis or investigating osteogenic differentiation of MSCs under mechanical strain [203,204,205,206,207,208]. According to their research question, the published microfluidic chips focus on certain physiological parameters that determine bone function in vivo such as hypoxia or mechanical load instead of a full recreation of bone biology (Figure 2).

The employed designs range from being rather simple to sophisticated depending on which aspect of bone biology is meant to be modeled. A simple cage made of polymethylmethacrylate (PMMA) filled with a hydrogel was proposed for investigating critical parameters that govern angiogenesis in bone (Figure 2a) [206]. In an attempt to create a vascularized bone tissue model, a modified version of an already published platform based on polydimethylsiloxane (PDMS) that was explicitly designed for investigating angiogenesis was proposed (Figure 2b). Here, microfluidic channels are partitioned by microposts to allow spatial separated co-culture of different cell types while leaving the possibility of sprouting in-between the different culture compartments [205]. The microfluidic perfusion culture of bone organoids was achieved either by a rather simple approach positioning the organoid within two layers of PDMS, or by incorporation in an already existing multi-organ chip [203,204]. Another approach aims to recreate the OCT network found in native bone by microfluidic perfusion of several layers made of HA microbeads intermingled with OCTs (Figure 2c). The microbeads and the cells are housed in a PDMS device and are mechanically stimulated in a cyclic manner through bidirectional changes in perfusion rate, an approach that recreates the shear stresses and hydrostatic pressures encountered in the canaliculi of bone [207]. In order to investigate the influence of mechanical strain on the osteogenic differentiation of MSCs from different sources, a microfluidic chip was designed that allows for pneumatic actuation of several culture chambers in parallel (Figure 2e). Three layers of PMMA were used to create the main body of the device while a flexible PDMS layer was incorporated to allow application of cyclic pneumatic force [208]. The employed systems exemplify how key aspects of bone physiology such as hypoxic conditions or the presence of mechanical forces can be modelled by microfluidic devices. Future designs might combine these approaches to generate more complex models of bone.

The materials used for the generation of ECMs in the mentioned microfluidic systems include demineralized bone powder for ectopic bone formation, HA microbeads, ceramic scaffolds and fibrin- or collagen-based hydrogels [203,204,205,206,207]. While ectopic formed bone does indeed resemble native bone in terms of cellular composition and ECM, it suffers from the disadvantage of relying on a human or animal donor, meaning that availability is limited while standardization would be hard to achieve (Figure 2d). Animal donors might also pose a problem when the bone-on-a-chip (BoC) is meant to be used as an alternative method to animal testing itself. The technique of ectopic bone formation in animals also prohibits the generation of fully humanized bone organoids, since it relies on the ingrowth of host cells. A ceramic scaffold does resemble the properties of cancellous bone in terms of stiffness and porosity, yet they are made of HA coated zirconium oxide, a material that barely resembles the composite of collagen and HA that is found in native bone. Although its mechanical properties in terms of stiffness resemble those of adult bone, it is unlikely to be a suitable substrate for successful remodeling activity by OCTs and OBs. A scaffold-free approach was chosen by Sun et al. [207] to allow the formation of cavities similar to the lacunae in native bone that house OCTs and their protrusions. Here, HA microbeads serve as the basis for the formation of an ECM [207]. Hydrogels as used by Bersini et al. can be made from recombinant proteins such as fibrin or collagen that allow for remodeling by cells but do not have the same mechanical properties as native adult bone [206]. In terms of cells used, MSCs seem to be the most popular ones used in the context of microfluidic bone models, due to their potential to differentiate into bone-forming OBs and their participation in the stromal part of the HSC niche [204,206,208]. The additional incorporation of HUVECs for subsequent vascularization was also proposed and ratio of a 10:1 (HUVECs:MSCs) was found to be optimal to generate bone-mimicking pre-vascularized hydrogels [206]. In another model, Jusoh and colleagues used HUVECs to vascularize a matrix made of fibrin and HA nanoparticles in order to generate a bone-like environment [205]. Yet this model lacks other cell types usually found in bone such as OBs, OCs, HSCs or MSCs. In the model of Sun and colleagues OBs and OCTS are used to investigate their interplay in response to mechanical stimulation that was facilitated by the application of shear stress [207]. Among published systems, two groups have reported the successful incorporation of HSCs and their progeny into their bone-on-a-chip (BoC) [203,204]. In the case of ectopic formed bone, the cells found in the organoid are exclusively of host (mouse) origin [203]. The use of cells isolated from human donors was presented by Sieber et al., who used bone marrow and umbilical cord blood for the isolation of MSCs and hematopoietic stem and progenitor cells (HSPCs) respectively [204]. In both cases an active haematopoiesis was demonstrated by the presence of differentiated immune cells. Furthermore, HSCs were kept in their native undifferentiated state, which is usually hard to achieve in vitro. Only Torisawa and colleagues reported active remodeling of their organoid as shown by the activity of OBs and OCs [203]. In comparison with the other BoCs, the system of Torisawa et al. recreates the cellular composition and ECM found in bone most accurately and was shown to enable the testing of drugs, for example for treatment of radiation damage [209]. The reviewed microfluidic systems are summarised in Supplementary Table S3.

## 6. Concluding Remarks

Due to its role in haematopoiesis, locomotion, organ protection and mineral homeostasis, tissue engineering bone in its complexity remains challenging. Therefore, most bone models do not try to recapitulate bone in its entirety but aspects of its biology and function that are important for the respective research question. Yet, it needs to be thoroughly reflected if the model of choice is complex enough to sufficiently represent the aspects of bone physiology under investigation. For example, remodeling requires the interaction of OBs and OCs and is guided by external signals such as mechanical load or parathyroid hormones in vivo. Thus, a system for investigating remodeling would need at least these two cell types and an external cue driving the process. Moreover, a suitable matrix would be of need, since the resorption of bone ECM by OCs is an enzyme and pH-driven process that is fine tuned to degrade the physiological components of bone matrix but not necessarily all scaffold materials available [24]. Haematopoiesis on the other hand is facilitated through HSCs and it is well described that maintaining the HSC phenotype including the stem cell properties is hard to achieve in cell culture [210]. In vivo, the HSC niche is formed by complex interactions with stromal and endothelial cells while the exact stem cell phenotype is also a function of local oxygen levels [12,19,58]. To date and to the best of our knowledge, two groups reported the presence of stromal cells and HSCs and successful haematopoiesis in their microfluidic models, providing proof-of-concept that the HSC niche can be indeed be rebuilt in organoids [200,201]. The increase in model complexity is thus not mandatory but allows for more sophisticated research aims without the use of whole organisms. It remains elusive if all aspects of bone physiology can be modelled in vitro. One current limitation is the inability to generate bone with comparable mechanical and anatomical properties. Although made of the same material, the different mechanical properties of cortical and cancellous bone stem from differences in orientation and alignment of collagen fibres that have not been recreated in vitro yet. Therefore, biomechanical investigations will be only approximations of what cells experience in vivo. Furthermore, the dense cortical bone and the sponge-like cancellous bone have dissimilar anatomical structures and vasculature that can’t be reproduced with current methods.

In addition to the choice of cells, the choice of a suitable ECM is vital for the fabrication of a bone organoid. A scaffold-free approach is in general favourable as it does not rely on artificial, xenogeneic or allogenic components and resembles the in vivo situation more closely in terms of ECM deposition and spatial organization. Nevertheless, these approaches are hard to standardize as they rely on self- organization of the utilized cells. Further, the scaffold-free generation of a bone-like ECM is a time-dependent process and will not instantly yield in an ECM with the same mechanical and biological properties found in vivo. Here, the use of a scaffold, in theory, can provide an ECM with the correct biological and mechanical properties at the time of deployment. The choice between scaffold-free and scaffold-based approaches is thus primarily a question of culture time and biomechanics. Another advantage of scaffold-free approaches is their similarity to developmental processes [211]. Scaffold-free approaches are suitable to model intramembranous or endochondral ossification, since they rely on condensation and subsequent matrix deposition of OBs or MSCs. This enables the establishment of model systems for the development of bones during embryogenesis [76,77,211]. Scaffolds can be made of a multitude of available materials with defined mechanical properties and might be further modified to guide cell differentiation, proliferation or migration. Yet it needs to be noted that the choice of scaffold material will always be a trade-off between different parameters and needs to be decided in regard of the models’ research question and purpose. For example, a ceramic scaffold made of zirconium that has the correct biomechanical properties might still be unsuited for questions regarding remodeling as it cannot be degraded by OCs.

The use of bioprinting for the generation of a multicellular bone organoid remains to be explored. However, a number of suitable bioinks have already been proposed and characterized. The parallel procession of multiple bioinks, a higher resolution and a bioink that resembles the ECM of native bone would be of tremendous advantage. Unlike all other approaches for the generation of bone organoids, 3D printing allows for the reproducible generation of anatomical features, thus facilitating the fabrication of standardized organoids with a desired anatomical structure.

Bioreactor systems can provide perfusion, mechanical actuation and gas exchange for more complex bone models. Among published bioreactors, some are able to provide these features, yet most of them are customized systems that rely on special hard- and software that is not commercially available. Furthermore, these systems seldom allow for high throughput applications. Besides their advantages, the main limitations of current bioreactor systems can be therefore summarized by their comparably low throughput, restricted user accessibility and the accompanying costs. Keeping these limitations in mind while profiting from the data generated with bioreactor systems, the development of microfluidic systems might be applicable to overcome existing restrictions. Classical 2D cell culture techniques on the other hand remain the standard in life sciences, since they are easy to handle, easy to manipulate, less sensitive to error, not overly complex and thus don’t require high technical skills. The rather laborious procedures associated with generating and culturing 3D organoids in a bioreactor system will always be a drawback when compared to 2D cultures as they hamper high-throughput applications and limit accessibility. Peripheral equipment with general purposes (e.g., pumps, sensor interfaces, microscopes) for the operation of microfluidic bioreactors offers the possibility to use commercially available devices. The chip itself is often disposable and might be lab-made by soft- lithography or mould casting, thus being one of the cheapest parts in a microfluidic setup. If the material of choice or the chip architecture requires the use of materials that need to be processed through injection moulding, the chip fabrication might be facilitated by specialized companies. The overall accessibility and of an OoC system is therefore increased when compared to custom made bioreactor systems. Since in vivo analyses are extremely difficult for bone and the corresponding cells, chip technology can enable new insights and gains in knowledge. And indeed, as summarized in this review, there are already systems published that recreate single key aspects of bone physiology, yet none of these combine them in one platform towards a physiologic bone-on-a-chip system.

**Figure 3:**
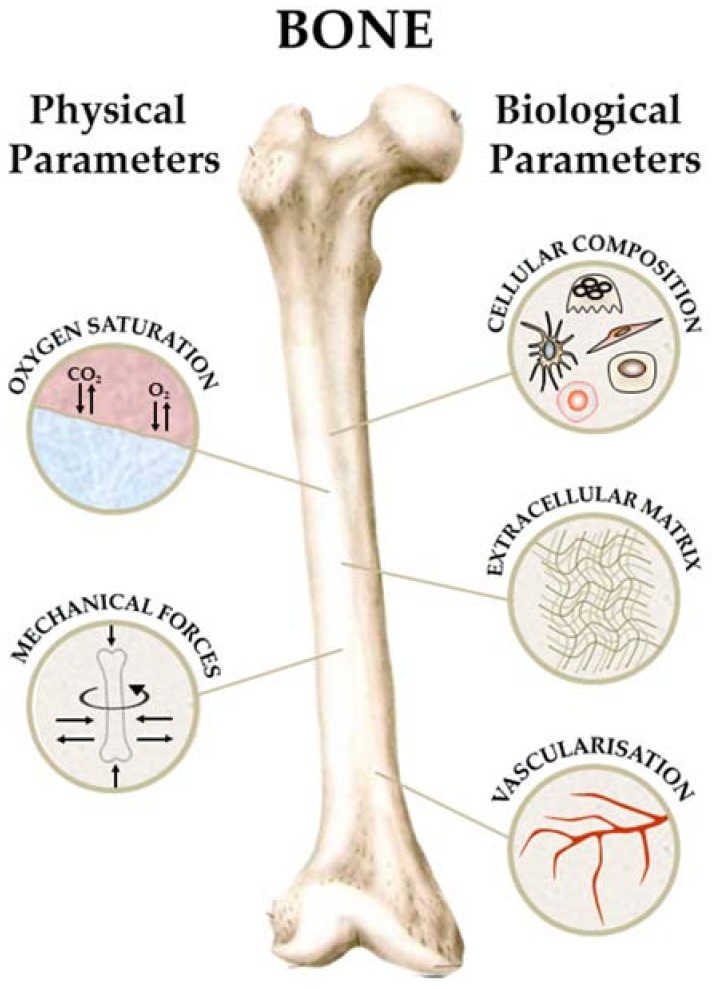
Schematic summary of key parameters in bone tissue.

The ultimate bone model would of course be physiologic bone itself. Yet, in light of current developments and literature we would like to propose a couple of considerations that we find to be key parameters in bone physiology (Figure 3). Depending on the research question, aspects of bone biology might be excluded in the model of choice. The model should allow for a limited control of physiochemical parameters in a user friendly manner while costs should be as low as possible. High parallelization should be feasible to allow for mid to high-throughput investigations. The model should exclude xenogeneic substances and materials, since these can have unintended side effects on the cells biology. A minimal set of cells that should be included would encompass MSCs, HSCs, OBs, OCs and if possible endothelial cells for vasculature. The ECM should ideally be made of materials that are of biological origin and resemble the native bone ECM as close as possible or might even be formed by scaffold-free approaches. Since mechanical load is present in bone throughout an organism’s lifetime and has a profound effect on bone cells, it should be included in the model. As most tissues in humans, bone is considered to be hypoxic and the influence of oxygen levels on local cells is well established. A physiologic bone model would therefore include regulation of oxygen levels to provide hypoxic growth conditions. Advances in cell isolation, culture and differentiation in combination with tissue engineering and microfluidics will likely enable the generation of advanced bone models that include some or even all aspects mentioned.

The importance of physiologic bone models will remain and maybe even increase due to several developments. The ever-increasing life expectancy due to advances in medicine will lead to an increase in the elderly population, thus musculoskeletal disorders and the development of treatments will be shifted even more in the focus of clinical research. Prominent disorders are osteoporosis or delayed fracture healing that still can only be treated symptomatically [1,212]. The development of successful treatments requires a profound understanding of the underlying pathologies. Reliable model systems are thus mandatory for advancements that are meant to counteract age-related bone loss and disease. Another result of a more elderly population is the increased use of joint implants to restore mobility, for hip joints the replacement procedure has already been dubbed the “operation of the century” [2]. It is well established that wear from the articulating surfaces of joint implants has detrimental local and systemic effects. Among these, bone loss around the implant can lead to loosening, thereby causing pain and mechanical instability. For example, metal-on-metal pairings were widely used for hip implants due to the low amounts of wear generated. It has however been demonstrated that these implants suffer from high failure rates due to the release of cobalt and chrome that shifts the tissue homeostasis in bone towards an increase in resorption while proper matrix mineralization is inhibited [213]. Further investigations were able to demonstrate that patients with hip implants are additionally exposed to a multitude of different metals such as titanium, vanadium and iron with unknown consequences for the patient [214]. To counteract the detrimental effects of metallic wear, different materials such as ceramics are introduced, yet their biological effects are poorly characterized. It needs to be noted that there are no reliable test systems available that allow for the investigation of biological effects of wear and corrosive products from implants. Here, complex microfluidic bone models that include remodeling might enable the testing of orthopaedic materials and their wear products prior application in humans.

Among future challenges for mankind, space exploration is becoming a realistic prospect since technical advancements have led to a significant drop in the associated costs. In this regard, the exploration of the solar systems is seriously discussed and planned with the Earth’s moon and Mars as the most likely first targets. While technology enables the construction of necessary space crafts, little is known about the long-term effects of zero gravity and cosmic radiation on the human body [215,216]. The only long-term data available was gathered on orbital space stations in microgravity. Yet, it has become evident that severe bone and muscle loss are among the changes that an astronaut encounters during space flight [217,218]. Unlike on Earth, physical exercise and therefore the application of additional mechanical load is not able to completely counteract bone loss experienced in extra-terrestrial space [219]. It remains therefore unclear if long-term space missions are feasible and if the bone loss encountered can be fully mitigated. In a worst case scenario, the loss of bone and muscles encountered during a mission spanning a couple of months or years (e.g., reaching Mars) would let the return to Earth’s gravity become a high risk operation for the astronaut’s health. Systems that are able to investigate the effect of micro- or zero gravity on bone formation would be therefore of high value. Fully automated microfluidic culture systems for bone cells have already found their way to the International Space Station [220]. Yet these systems do not recapitulate the most important aspect of bone physiology in this context that is remodeling in response to mechanical load. In analogy to the formulated ideal bone model, more sophisticated automated systems for investigations under micro or zero gravity would be of enormous value to find substances or loading regimes that reduce negative effects on bone density. The need for better bone models is thus not only rooted in the present but is mandated by foreseeable future developments.

## Figures and Tables

**Table 1:** **Table 1**. Hydrogels: Advantages and disadvantages.

Polymer Origin	Advantages	Disadvantages
Natural	Biocompatibility and their degradation is facilitated by enzymes present in vivo	Inconsistent hydration and elastic properties
Synthetic	Improved consistency and ability to modify properties (degradation, cell binding)	Weak mechanical strength and inability to sequester growth factors, resulting in burst release

## Supplementary Materials

The following are available online at http://www.mdpi.com/2073-4425/9/5/247/s1, Table S1: Overview on scaffold based bone models published between 2007 and 2017, Table S2: Summary of bioreactors suitable for bone models, Table S3: Summary of microfluidic bone models. References [221,222,223,224,225,226,227,228,229,230,231,232,233,234,235,236,237,238,239,240,241,242,243,244,245,246,247,248,249,250,251,252,253,254,255,256] are cited in the supplementary materials.
