# Supplementary material for: Journey into Bone Models: A Review
Source: Genes (Basel). 2018 May 10;9(5):247. doi: 10.3390/genes9050247 (PMC5977187; doi:10.3390/genes9050247)
Supplement: Supplementary file 1 [file genes-09-00247-s001.pdf]

Review

Journey into Bone Models: A Review

Julia Scheinpflug [1,†], Moritz Pfeiffenberger [2,3,†], Alexandra Damerau [2,3], Franziska Schwarz [1], Martin Textor [1], Annemarie Lang [2,3] and Frank Schulze [1,*]

[1] German Federal Institute for Risk Assessment (BfR), German Centre for the Protection of Laboratory Animals (Bf3R),10589 Berlin, Germany; julia.scheinpflug@bfr.bund.de (J.S.); franziskawschwarz@googlemail.com (F.S.); frank.schulze@bfr.bund.de (F.S.)

[2] Charité – Universitätsmedizin Berlin, corporate member of Freie Universität Berlin, Humboldt-Universität zu Berlin and Berlin Institute of Health, Department of Rheumatology and Clinical Immunology, 10117 Berlin, Germany; moritz.pfeiffenberger@charite.de (M.P.); alexandra.dameau@charite.de(A.D.); annemarie.lang@charite.de (A.L.)

[3] German Rheumatism Research Centre (DRFZ) Berlin, a Leibniz Institute,10117 Berlin, Germany

* Correspondence: frank.schulze@bfr.bund.de; Tel.: +49-30-18412-3702

† These authors contributed equally as first authors

Supplementary material

Table S1: Overview on scaffold-based bone models published between 2007 and 2017

Scaffold	Application in vitro / in vivo	Results	Field of application	References
**TCP or CaP-based scaffolds**				
β-TCP	monkey MSCs/ monkeys	β-TCP/MSC showed better bone regeneration compared to β-TCP	in vitro model and BTE application	[1]
HA/TCP	human osteoblasts / -	adhesion, proliferation, osteogenesis	in vitro model	[2]
apatite-TCP, HA	mouse calvarial cells / -	homogeneous loading, enhancing its mechanical strength, cells grow and differentiate as osteoblasts in a reproducible manner	in vitro system to analyze bone cell function and bone-targeting molecules under load	[3,4]
mPCL/TCP	sheep MPC and OB/ sheep	higher bone formation with MPCs, allogenic cells induced no acute or delayed immunological response	in vitro model and BTE application	[5,6]
TCP-BG/PLL		high amount of carbonate apatite, better bioactivity properties compare to PLL	in vitro model and BTE application	[7,8]
PLG/TCP/Icariin	MC3T3-E1 osteoblastic cells (Subclone 14, CRL-2594) / SAON rabbit	superior biodegradability, biocompatibility, and osteogenic ability compared to PLG/TCP	in vitro model and BTE application focused on SAON	[9]
CHS/CaP, CHS/CaP/Pg	murine MSCs / male albino Wistar rats	Pigeonite enhances bioactivity, biomineralization and osteoblast differentiation	in vitro model and BTE application	[10]
CDHA, biphasic CaP (80:20 HA:β-TCP), β-TCP	rat MSCs / canine	CDHA: accelerated bone formation and new ectopic bone replaced scaffold; CaP: bone deposited on the surface	in vitro model and BTE application	[11]

Table S1. *Cont.*

Scaffold		Application in vitro / in vivo	**Table 1.** *Cont.*	Field of application
**Natural polymer-based scaffolds**				
collagen loaded with secretome biotherapeutic	rat MSCs, rat calvarial osteoprogenitor cells / rat	enhanced migration, proliferation, new bone volume, connectivity, and angiogenesis	beneficial effects for bone healing in vivo	[12]
collagen/β-TCP, collagen	rat MSCs / -	collagen/β-TCP > collagen: osteogenesis, attached cell number, expression of osteogenic markers	in vitro model and BTE application	[13,14]
fibrin glue scaffold	equine MSCs / mouse	osteogenic differentiation capacity	in vitro model and BTE application	[15]
CHS, PGel, CS-PGel	- / rat	*in vivo* bone regeneration: CHS < CHS-PGel < PGel	in vitro model and BTE application	[16–19]
recombinant human BMP-2-loaded gelatin/nano-HA/fibrin	human MSCs / rabbit	osteogenic capability	in vitro and in vivo: growth-factor delivery carrier and a 3D matrix	[20]
recombinant human BMP-2-loaded CHS/ collagen	- / rabbit	biocompatibility, bone formation, appropriate degradation	Scaffold-mediated drug delivery for BTE, treating segmental bone defects	[21,22]
CHS/nano-HA/SF	MSCs / rabbit	in vitro: biocompatibility, no toxicity, cell adhesion and proliferation; in vivo: new bone	in vitro model and BTE application to repair segmental defect	[23,24]
substance-doped HA	MG63 human osteoblasts cells	Lithium promote osteoblast activity; degradation rate of LiHA < HA	in vitro model and BTE application	[25–27]
HA/alumina scaffold	- / canine	scaffold with a 3mm passage formed new bone compared to the scaffold without a passage	in vitro model and BTE application	[28]
CHS/ β-1,3-glucan /HA	human AT- and marrow-derived MSCs / -	both cells proliferate and differentiate, bone marrow-derived spread better and attached stronger	in vitro model and BTE application	[29–31]
nano-HA/polyamide 66	rat MSCs / -	sufficient proliferation and differentiation greatly improve through perfusion culture conditions	in vitro model and BTE application	[32]
TGF-β1-SF-CHS, SF-CHS	rabbit MSCs / rabbit	TGF-β1-SF-CHS: biocompatibility, enhance osteoconductivity and bone formation	in vitro model and BTE application	[33]
ASA scaffold, ASA/β-TCP scaffold	canine MSCs / canine	higher bone formation with ASA scaffold compared to ASA/β-TCP	in vitro model and BTE application	[34]
Calcium-Infiltrated HA	HUVECs / -	calcium release at the surface, promoted a hematopoietic lineage direction of HUVECs	in vivo-like scaffold for hematopoietic BTE	[35]

Synthetic polymer-based scaffolds

PLG-mediated minicircle DNA (MC) delivery	Skull-derived osteoblasts transfected with BMP-2 / mice	higher osteocalcin expression and mineralization	Scaffold-mediated gene delivery for BTE, treating long bone defects	[36]
PCL/PLG, PCL/PLG/duck beak, PCL/PLG/TCP	- / rabbit	bone formation: PCL/PLG < PCL/PLG/TCP < PCL/PLG/duck beak	in vitro model and BTE application	[37]
polydopamine-coated PLG-[Asp-PEG]	rat MSCs / rat	could more efficiently promote osteogenic differentiation *in vitro*	-	[38]
PLG/PEG with incorporated BMP-2	human MSCs / mouse	osteogenesis, bone formation	in vitro model and BTE application	[39,40]
(30:70% *w/w*) SPCL, SPCL-Si	- / rat	SPCL-Si: significant higher bone formation	in vitro model and BTE application	[41]
nano-HA/PLG, PLGA	rabbit MSCs / rabbit	Viability and proliferation rate, bone formation rate: nano-HA/PLGA > PLGA	in vitro model and BTE application	[27,42,43]
(60:40) PCL/HA, PCL	canine MSCs / canine	bone regeneration, seeded with MSCs enhance the amount of bone ingrowth	in vitro model and BTE application	[39,44–48]

Table S2: Summary of bioreactors suitable for bone models

Scaffold	Cells	Vascularisation	Mech. load	Perfusion	Reference
physiologic matrix (explant)	multiple (whole bone explant)	yes (explant)	yes	yes	[49]
hydroxyapatite ceramic	human MSCs, HSCs	no	no	yes	[50]
hydroxyapatite ceramic	human MSCs (primary isolates)	no	no	yes	[51]
trabecular bone (bovine), 3D CNC milled	human MSCs (primary isolates)	no	no	yes	[52]
poly (L-lactide-co-caprolactone)	human MSCs (primary isolates)	no	no	yes	[53]
polyurethane	Fibroblasts (commercial, ATCC)	no	yes	no	[54]
commercial animal derived collagen scaffold, NiTi scaffold	human MSCs (primary isolates)	no	yes	yes	[55]
hydroxyapatite ceramic	human MSCs and stromal vascular fraction cells (both primary isolates)	yes	no	yes	[56]
hydroxyapatite ceramic	whole mononuclear fraction isolated from bone marrow (primary isolate, commercial)	no	no	yes	[57]
collagen scaffold	fibroblasts (primary isolates)	no	yes	yes	[58]
acellular bone matrix (bovine)	human MSCs (primary isolates)	no	yes	yes	[59]
fibrin	human MSCs (primary isolates)	no	yes	yes	[60]

Table S3: Summary of microfluidic bone models

Scaffold	Cells	Mech. load	Vascularisation	Perfusion	Chip material	Reference
collagen and fibrinogen hydrogel	hMSC, hMSC-OB (primary isolates), HUVECs (commercial, transfected primary cells)	no	yes	yes	PMMA	[61]
fibrin with hydroxy apatite NPs	HUVECs (undisclosed)	no	yes	yes	PDMS	[62]
none	hMSCs (commercial, primary isolates)	yes (stretchin)	no	yes	PDMS, PMMA, glass	[63]
demineralized bone powder	animal, bone-inducing material is pellettet and subcutanously implanted for host cell ingrowth	no	yes	yes	PDMS	[64]
ceramic, hydroxyapatite coated zirconium oxide	hMSC, HSPCs (primary isolates)	no	no	yes	PDMS, glass	[65]
Hydroxyapatite beads (20-25 μm in size)	MLO-A5 (post-osteoblast/pre-osteocyte cell line), hOB (primary isolate)	yes (shear)	no	yes	PDMS	[66]

References

1. Masaoka, T.; Yoshii, T.; Yuasa, M.; Yamada, T.; Taniyama, T.; Torigoe, I.; Shinomiya, K.; Okawa, A.; Morita, S.; Sotome, S. Bone defect regeneration by a combination of a β-tricalcium phosphate scaffold and bone marrow stromal cells in a non-human primate model. *Open Biomed. Eng. J.* **2016**, *10*, 2–11.
2. Almela, T.; Al-Sahaf, S.; Bolt, R.; Brook, I.M.; Moharamzadeh, K. Characterization of multilayered tissue-engineered human alveolar bone and gingival mucosa. *Tissue Eng. Part C Methods* **2018**.
3. Bouet, G.; Cruel, M.; Laurent, C.; Vico, L.; Malaval, L.; Marchat, D. Validation of an in vitro 3D bone culture model with perfused and mechanically stressed ceramic scaffold. *Eur. Cell Mater.* **2015**, *29*, 250–266; discussion 266–257.
4. Tanaka, M.; Haniu, H.; Kamanaka, T.; Takizawa, T.; Sobajima, A.; Yoshida, K.; Aoki, K.; Okamoto, M.; Kato, H.; Saito, N. Physico-chemical, in vitro, and in vivo evaluation of a 3D unidirectional porous hydroxyapatite scaffold for bone regeneration. *Materials (Basel)* **2017**, *10*.
5. Baykan, E.; Koc, A.; Eser Elcin, A.; Murat Elcin, Y. Evaluation of a biomimetic poly(epsilon-caprolactone)/beta-tricalcium phosphate multispiral scaffold for bone tissue engineering: In vitro and in vivo studies. *Biointerphases* **2014**, *9*, 029011.
6. Berner, A.; Henkel, J.; Woodruff, M.A.; Saifzadeh, S.; Kirby, G.; Zaiss, S.; Gohlke, J.; Reichert, J.C.; Nerlich, M.; Schuetz, M.A.; et al. Scaffold-cell bone engineering in a validated preclinical animal model: Precursors vs differentiated cell source. *J Tissue Eng. Regen. Med.* **2017**, *11*, 2081–2089.
7. Ghasemi, A.; Hashemi, B. Co-existence effect of tricalcium phosphate and bioactive glass on biological and biodegradation characteristic of poly l-lactic acid (PLLA) in trinary composite scaffold form. *Biomed. Mater. Eng.* **2017**, *28*, 655–669.
8. Nadeem, D.; Kiamehr, M.; Yang, X.; Su, B. Fabrication and in vitro evaluation of a sponge-like bioactive-glass/gelatin composite scaffold for bone tissue engineering. *Mater. Sci. Eng. C Mater. Biol. Appl.* **2013**, *33*, 2669–2678.
9. Lai, Y.; Cao, H.; Wang, X.; Chen, S.; Zhang, M.; Wang, N.; Yao, Z.; Dai, Y.; Xie, X.; Zhang, P.; et al. Porous composite scaffold incorporating osteogenic phytomolecule icariin for promoting skeletal regeneration in challenging osteonecrotic bone in rabbits. *Biomaterials* **2018**, *153*, 1-13.
10. Dhivya, S.; Keshav Narayan, A.; Logith Kumar, R.; Viji Chandran, S.; Vairamani, M.; Selvamurugan, N. Proliferation and differentiation of mesenchymal stem cells on scaffolds containing chitosan, calcium polyphosphate and pigeonite for bone tissue engineering. *Cell. Prolif.* **2017**.
11. Barba, A.; Diez-Escudero, A.; Maazouz, Y.; Rappe, K.; Espanol, M.; Montufar, E.B.; Bonany, M.; Sadowska, J.M.; Guillem-Marti, J.; Ohman-Magi, C.; et al. Osteoinduction by foamed and 3D-printed calcium phosphate scaffolds: Effect of nanostructure and pore architecture. *ACS Appl. Mater. Interfaces* **2017**, *9*, 41722–41736.
12. Burdette, A.J.; Guda, T.; Thompson, M.E.; Banas, R.; Sheppard, F. A novel secretome biotherapeutic influences regeneration in critical size bone defects. *J. Craniofac. Surg* **2018**, *29*, 116–123.
13. Todo, M.; Arahira, T. In vitro bone formation by mesenchymal stem cells with 3D collagen/beta-tcp composite scaffold. *Conf. Proc. IEEE Eng. Med. Biol. Soc.* **2013**, *2013*, 409–412.
14. Xie, L.; Zhang, N.; Marsano, A.; Vunjak-Novakovic, G.; Zhang, Y.; Lopez, M.J. In vitro mesenchymal trilineage differentiation and extracellular matrix production by adipose and bone marrow derived adult equine multipotent stromal cells on a collagen scaffold. *Stem Cell Rev.* **2013**, *9*, 858–872.
15. McDuffee, L.A.; Esparza Gonzalez, B.P.; Nino-Fong, R.; Aburto, E. Evaluation of an in vivo heterotopic model of osteogenic differentiation of equine bone marrow and muscle mesenchymal stem cells in fibrin glue scaffold. *Cell Tissue Res.* **2014**, *355*, 327–335.
16. Chen, Y.; Liu, X.; Liu, R.; Gong, Y.; Wang, M.; Huang, Q.; Feng, Q.; Yu, B. Zero-order controlled release of BMP2-derived peptide P24 from the chitosan scaffold by chemical grafting modification technique for promotion of osteogenesis in vitro and enhancement of bone repair in vivo. *Theranostics* **2017**, *7*, 1072–1087.
17. Dinescu, S.; Ionita, M.; Pandele, A.M.; Galateanu, B.; Iovu, H.; Ardelean, A.; Costache, M.; Hermenean, A. In vitro cytocompatibility evaluation of chitosan/graphene oxide 3D scaffold composites designed for bone tissue engineering. *Biomed. Mater. Eng.* **2014**, *24*, 2249–2256.
18. Li, Q.; Yu, X.; Zhou, G.; Wu, Y.W.; Hu, H.C.; Wang, T.; Tang, Z.H. [synthesis and in vitro characterization of chitosan microspheres/ceramic bovine bone composite scaffold]. *Beijing Da Xue Xue Bao* **2016**, *48*, 1043–1048.

19. Oryan, A.; Alidadi, S.; Bigham-Sadegh, A.; Moshiri, A. Effectiveness of tissue engineered based platelet gel embedded chitosan scaffold on experimentally induced critical sized segmental bone defect model in rat. *Injury* **2017**, *48*, 1466–1474.
20. Liu, Y.; Lu, Y.; Tian, X.; Cui, G.; Zhao, Y.; Yang, Q.; Yu, S.; Xing, G.; Zhang, B. Segmental bone regeneration using an rhBMP-2-loaded gelatin/nanohydroxyapatite/fibrin scaffold in a rabbit model. *Biomaterials* **2009**, *30*, 6276–6285.
21. Hou, J.; Wang, J.; Cao, L.; Qian, X.; Xing, W.; Lu, J.; Liu, C. Segmental bone regeneration using rhBMP-2-loaded collagen/chitosan microspheres composite scaffold in a rabbit model. *Biomed. Mater.* **2012**, *7*, 035002.
22. Qian, Y.; Lin, Z.; Chen, J.; Fan, Y.; Davey, T.; Cake, M.; Day, R.; Dai, K.; Xu, J.; Zheng, M. Natural bone collagen scaffold combined with autologous enriched bone marrow cells for induction of osteogenesis in an ovine spinal fusion model. *Tissue Eng. Part. A* **2009**, *15*, 3547–3558.
23. Gholipourmalekabadi, M.; Mozafari, M.; Bandehpour, M.; Salehi, M.; Sameni, M.; Caicedo, H.H.; Mehdipour, A.; Hamidabadi, H.G.; Samadikuchaksaraei, A.; Ghanbarian, H. Optimization of nanofibrous silk fibroin scaffold as a delivery system for bone marrow adherent cells: In vitro and in vivo studies. *Biotechnol. Appl. Biochem.* **2015**, *62*, 785–794.
24. L. Ramos, T.; Sánchez-Abarca, L.I.; Muntión, S.; Preciado, S.; Puig, N.; López-Ruano, G.; Hernández-Hernández, Á.; Redondo, A.; Ortega, R.; Rodríguez, C.; et al. MSC surface markers (CD44, CD73, and CD90) can identify human msc-derived extracellular vesicles by conventional flow cytometry. *J. Cell Commun. Signal* **2016**, *14*, 2.
25. Cakmak, S.; Cakmak, A.S.; Gumusderelioglu, M. Rgd-bearing peptide-amphiphile-hydroxyapatite nanocomposite bone scaffold: An in vitro study. *Biomed. Mater.* **2013**, *8*, 045014.
26. Carpena, N.T.; Min, Y.K.; Lee, B.T. Improved in vitro biocompatibility of surface-modified hydroxyapatite sponge scaffold with gelatin and BMP-2 in comparison against a commercial bone allograft. *ASAIO J.* **2015**, *61*, 78–86.
27. Wang, D.X.; He, Y.; Bi, L.; Qu, Z.H.; Zou, J.W.; Pan, Z.; Fan, J.J.; Chen, L.; Dong, X.; Liu, X.N.; et al. Enhancing the bioactivity of Poly (lactic-co-glycolic acid) scaffold with a nano-hydroxyapatite coating for the treatment of segmental bone defect in a rabbit model. *Int. J. Nanomed.* **2013**, *8*, 1855–1865.
28. Kim, J.M.; Son, J.S.; Kang, S.S.; Kim, G.; Choi, S.H. Bone regeneration of hydroxyapatite/alumina bilayered scaffold with 3 mm passage-like medullary canal in canine tibia model. *Biomed. Res. Int.* **2015**, 235108.
29. Koc, A.; Finkenzeller, G.; Elcin, A.E.; Stark, G.B.; Elcin, Y.M. Evaluation of adenoviral vascular endothelial growth factor-activated chitosan/hydroxyapatite scaffold for engineering vascularized bone tissue using human osteoblasts: In vitro and in vivo studies. *J. Biomater. Appl.* **2014**, *29*, 748-760.
30. Przekora, A.; Ginalska, G. In vitro evaluation of the risk of inflammatory response after chitosan/HA and chitosan/β-1,3-glucan/HA bone scaffold implantation. *Mater. Sci. Eng. C Mater. Biol. Appl.* **2016**, *61*, 355–361.
31. Przekora, A.; Vandrovcova, M.; Travnickova, M.; Pajorova, J.; Molitor, M.; Ginalska, G.; Bacakova, L. Evaluation of the potential of chitosan/β-1,3-glucan/hydroxyapatite material as a scaffold for living bone graft production in vitro by comparison of ADSC and BMDSC behaviour on its surface. *Biomed. Mater.* **2017**, *12*, 015030.
32. Qian, X.; Yuan, F.; Zhimin, Z.; Anchun, M. Dynamic perfusion bioreactor system for 3D culture of rat bone marrow mesenchymal stem cells on nanohydroxyapatite/polyamide 66 scaffold in vitro. *J. Biomed. Mater. Res. Part B Appl. Biomater.* **2013**, *101*, 893–901.
33. Tong, S.; Xu, D.P.; Liu, Z.M.; Du, Y.; Wang, X.K. Synthesis of and in vitro and in vivo evaluation of a novel TGF-β1-SF-CS three-dimensional scaffold for bone tissue engineering. *Int J. Mol. Med.* **2016**, *38*, 367–380.
34. Yoon, D.; Kang, B.J.; Kim, Y.; Lee, S.H.; Rhew, D.; Kim, W.H.; Kweon, O.K. Effect of serum-derived albumin scaffold and canine adipose tissue-derived mesenchymal stem cells on osteogenesis in canine segmental bone defect model. *J. Vet. Sci.* **2015**, *16*, 397–404.
35. Zhang, Q.; Gerlach, J.C.; Schmelzer, E.; Nettleship, I. Effect of calcium-infiltrated hydroxyapatite scaffolds on the hematopoietic fate of human umbilical vein endothelial cells. *J. Vasc. Res.* **2017**, *54*, 376–385.
36. Keeney, M.; Chung, M.T.; Zielins, E.R.; Paik, K.J.; McArdle, A.; Morrison, S.D.; Ransom, R.C.; Barbhaiya, N.; Atashroo, D.; Jacobson, G.; et al. Scaffold-mediated BMP-2 minicircle DNA delivery accelerated bone repair in a mouse critical-size calvarial defect model. *J. Biomed. Mater. Res. A* **2016**, *104*, 2099–2107.
37. Lee, J.Y.; Son, S.J.; Son, J.S.; Kang, S.S.; Choi, S.H. Bone-healing capacity of PCL/PLGA/Duck beak scaffold in critical bone defects in a rabbit model. *Biomed. Res. Int.* **2016**, *2016*, 2136215.

38. Pan, H.; Zheng, Q.; Guo, X.; Wu, Y.; Wu, B. Polydopamine-assisted BMP-2-derived peptides immobilization on biomimetic copolymer scaffold for enhanced bone induction in vitro and in vivo. *Colloids Surf. B Biointerfaces.* **2016**, *142*, 1–9.
39. Kang, S.W.; Bae, J.H.; Park, S.A.; Kim, W.D.; Park, M.S.; Ko, Y.J.; Jang, H.S.; Park, J.H. Combination therapy with BMP-2 and BMSCS enhances bone healing efficacy of PCL scaffold fabricated using the 3D plotting system in a large segmental defect model. *Biotechnol. Lett.* **2012**, *34*, 1375–1384.
40. Rahman, C.V.; Ben-David, D.; Dhillon, A.; Kuhn, G.; Gould, T.W.; Muller, R.; Rose, F.R.; Shakesheff, K.M.; Livne, E. Controlled release of BMP-2 from a sintered polymer scaffold enhances bone repair in a mouse calvarial defect model. *J. Tissue Eng. Regen. Med.* **2014**, *8*, 59–66.
41. Requicha, J.F.; Moura, T.; Leonor, I.B.; Martins, T.; Munoz, F.; Reis, R.L.; Gomes, M.E.; Viegas, C.A. Evaluation of a starch-based double layer scaffold for bone regeneration in a rat model. *J. Orthop. Res.* **2014**, *32*, 904–909.
42. Liang, H.; Wang, K.; Shimer, A.L.; Li, X.; Balian, G.; Shen, F.H. Use of a bioactive scaffold for the repair of bone defects in a novel reproducible vertebral body defect model. *Bone* **2010**, *47*, 197-204.
43. Shi, J.; Zhang, X.; Zeng, X.; Zhu, J.; Pi, Y.; Zhou, C.; Ao, Y. One-step articular cartilage repair: Combination of in situ bone marrow stem cells with cell-free Poly(l-lactic-co-glycolic acid) scaffold in a rabbit model. *Orthopedics* **2012**, *35*, e665–671.
44. Bhattacharjee, P.; Maiti, T.K.; Bhattacharya, D.; Nandi, S.K. Effect of different mineralization processes on in vitro and in vivo bone regeneration and osteoblast-macrophage cross-talk in co-culture system using dual growth factor mediated non-mulberry silk fibroin grafted poly (capital je, ukrainian-caprolactone) nanofibrous scaffold. *Colloids Surf. B Biointerfaces* **2017**, *156*, 270–281.
45. Suliman, S.; Xing, Z.; Wu, X.; Xue, Y.; Pedersen, T.O.; Sun, Y.; Doskeland, A.P.; Nickel, J.; Waag, T.; Lygre, H.; et al. Release and bioactivity of bone morphogenetic protein-2 are affected by scaffold binding techniques in vitro and in vivo. *J. Control. Release* **2015**, *197*, 148–157.
46. Vergroesen, P.P.; Kroeze, R.J.; Helder, M.N.; Smit, T.H. The use of Poly(l-lactide-co-caprolactone) as a scaffold for adipose stem cells in bone tissue engineering: Application in a spinal fusion model. *Macromol. Biosci.* **2011**, *11*, 722–730.
47. Veronesi, F.; Giavaresi, G.; Guarino, V.; Raucci, M.G.; Sandri, M.; Tampieri, A.; Ambrosio, L.; Fini, M. Bioactivity and bone healing properties of biomimetic porous composite scaffold: In vitro and in vivo studies. *J. Biomed. Mater. Res. A* **2015**, *103*, 2932–2941.
48. Xuan, Y.; Tang, H.; Wu, B.; Ding, X.; Lu, Z.; Li, W.; Xu, Z. A specific groove design for individualized healing in a canine partial sternal defect model by a polycaprolactone/hydroxyapatite scaffold coated with bone marrow stromal cells. *J. Biomed. Mater. Res. A* **2014**, *102*, 3401–3408.
49. Richards, R.G.; Simpson, A.E.; Jaehn, K.; Furlong, P.I.; Stoddart, M.J. Establishing a 3D ex vivo culture system for investigations of bone metabolism and biomaterial interactions. *ALTEX.* **2007**, *24*, 56–59.
50. Braccini, A.; Wendt, D.; Jaquiery, C.; Jakob, M.; Heberer, M.; Kenins, L.; Wodnar-Filipowicz, A.; Quarto, R.; Martin, I. Three-dimensional perfusion culture of human bone marrow cells and generation of osteoinductive grafts. *Stem Cells* **2005**, *23*, 1066–1072.
51. Wendt, D.; Marsano, A.; Jakob, M.; Heberer, M.; Martin, I. Oscillating perfusion of cell suspensions through three-dimensional scaffolds enhances cell seeding efficiency and uniformity. *Biotechnol. Bioeng.* **2003**, *84*, 205–214.
52. Grayson, W.L.; Frohlich, M.; Yeager, K.; Bhumiratana, S.; Chan, M.E.; Cannizzaro, C.; Wan, L.Q.; Liu, X.S.; Guo, X.E.; Vunjak-Novakovic, G. Engineering anatomically shaped human bone grafts. *Proc. Natl. Acad. Sci. U.S.A.* **2010**, *107*, 3299–3304.
53. Kleinhans, C.; Mohan, R.R.; Vacun, G.; Schwarz, T.; Haller, B.; Sun, Y.; Kahlig, A.; Kluger, P.; Finne-Wistrand, A.; Walles, H.; et al. A perfusion bioreactor system efficiently generates cell-loaded bone substitute materials for addressing critical size bone defects. *Biotechnol. J.* **2015**, *10*, 1727–1738.
54. Lasher, R.A.; Wolchok, J.C.; Parikh, M.K.; Kennedy, J.P.; Hitchcock, R.W. Design and characterization of a modified T-flask bioreactor for continuous monitoring of engineered tissue stiffness. *Biotechnol. Prog.* **2010**, *26*, 857–864.
55. Hoffmann, W.; Feliciano, S.; Martin, I.; de Wild, M.; Wendt, D. Novel perfused compression bioreactor system as an in vitro model to investigate fracture healing. *Front. Bioeng. Biotechnol.* **2015**, *3*, 10.

56. Scherberich, A.; Galli, R.; Jaquiery, C.; Farhadi, J.; Martin, I. Three-dimensional perfusion culture of human adipose tissue-derived endothelial and osteoblastic progenitors generates osteogenic constructs with intrinsic vascularization capacity. *Stem Cells* **2007**, *25*, 1823–1829.
57. Schmelzer, E.; Finoli, A.; Nettleship, I.; Gerlach, J.C. Long-term three-dimensional perfusion culture of human adult bone marrow mononuclear cells in bioreactors. *Biotechnol. Bioeng.* **2015**, *112*, 801–810.
58. Petersen, A.; Joly, P.; Bergmann, C.; Korus, G.; Duda, G.N. The impact of substrate stiffness and mechanical loading on fibroblast-induced scaffold remodeling. *Tissue Eng. Pt. A* **2012**, *18*, 1804–1817.
59. Jagodzinski, M.; Breitbart, A.; Wehmeier, M.; Hesse, E.; Haasper, C.; Krettek, C.; Zeichen, J.; Hankemeier, S. Influence of perfusion and cyclic compression on proliferation and differentiation of bone marrow stromal cells in 3-dimensional culture. *J. Biomech.* **2008**, *41*, 1885–1891.
60. Matziolis, D.; Tuischer, J.; Matziolis, G.; Kasper, G.; Duda, G.; Perka, C. Osteogenic predifferentiation of human bone marrow-derived stem cells by short-term mechanical stimulation. *Open Orthop. J.* **2011**, *5*, 1–6.
61. Bersini, S.; Gilardi, M.; Arrigoni, C.; Talo, G.; Zamai, M.; Zagra, L.; Caiolfa, V.; Moretti, M. Human in vitro 3D co-culture model to engineer vascularized bone-mimicking tissues combining computational tools and statistical experimental approach. *Biomaterials* **2016**, *76*, 157–172.
62. Jusoh, N.; Oh, S.; Kim, S.; Kim, J.; Jeon, N.L. Microfluidic vascularized bone tissue model with hydroxyapatite-incorporated extracellular matrix. *Lab. Chip* **2015**, *15*, 3984–3988.
63. Park, S.H.; Sim, W.Y.; Min, B.H.; Yang, S.S.; Khademhosseini, A.; Kaplan, D.L. Chip-based comparison of the osteogenesis of human bone marrow- and adipose tissue-derived mesenchymal stem cells under mechanical stimulation. *Plos ONE* **2012**, *7*.
64. Torisawa, Y.S.; Spina, C.S.; Mammoto, T.; Mammoto, A.; Weaver, J.C.; Tat, T.; Collins, J.J.; Ingber, D.E. Bone marrow-on-a-chip replicates hematopoietic niche physiology in vitro. *Nat. Methods* **2014**, *11*, 663-+.
65. Sieber, S.; Wirth, L.; Cavak, N.; Koenigsmark, M.; Marx, U.; Lauster, R.; Rosowski, M. Bone marrow-on-a-chip: Long-term culture of human haematopoietic stem cells in a three-dimensional microfluidic environment. *J. Tissue Eng. Regen. Med.* **2017**.
66. Sun, Q.L.; Choudhary, S.; Mannion, C.; Kissin, Y.; Zilberberg, J.; Lee, W.Y. Ex vivo replication of phenotypic functions of osteocytes through biomimetic 3D bone tissue construction. *Bone* **2018**, *106*, 148–155.
